# Frequent and Simultaneous Epigenetic Inactivation of *TP53* Pathway Genes in Acute Lymphoblastic Leukemia

**DOI:** 10.1371/journal.pone.0017012

**Published:** 2011-02-28

**Authors:** Amaia Vilas–Zornoza, Xabier Agirre, Vanesa Martín-Palanco, José Ignacio Martín-Subero, Edurne San José-Eneriz, Leire Garate, Sara Álvarez, Estíbaliz Miranda, Paula Rodríguez-Otero, José Rifón, Antonio Torres, María José Calasanz, Juan Cruz Cigudosa, José Román-Gómez, Felipe Prósper

**Affiliations:** 1 Hematology Service and Area of Cell Therapy, Clínica Universidad de Navarra, Foundation for Applied Medical Research, University of Navarra, Pamplona, Spain; 2 Hematology Department, Reina Sofia Hospital, Maimonides Institute for Biomedical Research, Cordoba, Spain; 3 Department of Anatomic Pathology, Pharmacology and Microbiology, University of Barcelona, Barcelona, Spain; 4 Molecular Cytogenetics Group, Centro Nacional Investigaciones Oncológicas (CNIO), Centro de Investigaciones de Enfermedades Raras (CIBERER), Madrid, Spain; 5 Department of Genetics, University of Navarra, Navarra, Spain; Bellvitge Biomedical Research Institute (IDIBELL), Spain

## Abstract

Aberrant DNA methylation is one of the most frequent alterations in patients with Acute Lymphoblastic Leukemia (ALL). Using methylation bead arrays we analyzed the methylation status of 807 genes implicated in cancer in a group of ALL samples at diagnosis (n = 48). We found that 154 genes were methylated in more than 10% of ALL samples. Interestingly, the expression of 13 genes implicated in the *TP53* pathway was downregulated by hypermethylation. Direct or indirect activation of *TP53* pathway with 5-aza-2′-deoxycitidine, Curcumin or Nutlin-3 induced an increase in apoptosis of ALL cells. The results obtained with the initial group of 48 patients was validated retrospectively in a second cohort of 200 newly diagnosed ALL patients. Methylation of at least 1 of the 13 genes implicated in the *TP53* pathway was observed in 78% of the patients, which significantly correlated with a higher relapse (p = 0.001) and mortality (p<0.001) rate being an independent prognostic factor for disease-free survival (DFS) (p = 0.006) and overall survival (OS) (p = 0.005) in the multivariate analysis. All these findings indicate that *TP53* pathway is altered by epigenetic mechanisms in the majority of ALL patients and correlates with prognosis. Treatments with compounds that may reverse the epigenetic abnormalities or activate directly the p53 pathway represent a new therapeutic alternative for patients with ALL.

## Introduction

Acute lymphoblastic leukemia (ALL), the most common kind of cancer in children, is associated with a number of genetic lesions responsible for impairment of normal cellular behavior. Chromosomal translocations identify unique subtypes of the disease [1], and although detected in less than 50% of patients with B-ALL and in a significantly lower percentage of T-ALL, they have been associated with specific prognostic groups [2]. These translocations usually activate transcription factors responsible for the control of cell differentiation, proliferation and apoptosis [3]. Recent evidence suggests that translocations act in concert with other genetic lesions to induce overt leukemia including deletion of genes such as cyclin-dependent kinase inhibitor 2A gene (CDKN2A) [4] or the more recently described deletion of *IKZF1* (Ikaros) [5].

Unlike solid tumors, point mutations involving oncogenes or tumor suppressor genes are rarely seen in patients with ALL [6]. *TP53* is one of the genes most frequently mutated in cancer, with inactivating mutations present in over 50% of patients with solid tumors. However, less than 3% of patients with ALL show mutations of *TP53*, despite the fact that ALL cells present an abnormal resistance to apoptosis, a hallmark of deregulated p53 pathway [7]. The low percentage of *TP53* mutations in ALL patients as well as the resistance to apoptosis suggest that regulation of apoptosis in ALL could be mediated by other mechanisms involved in the abnormal function of the p53 pathway or p53 independent mechanisms, including the deregulation of genes of the BCL2 family such as *BCL2, BAX, BCL-XL*, mechanisms that should be explored in order to improve our understanding of the pathogenesis of the disease

The role of aberrant epigenetic modifications in cancer development and particularly in hematological malignancies such as acute leukemias and myelodysplastic syndromes has clearly been recognized in the last years [8], [9]. Several groups have demonstrated that the abnormal methylation of multiple genes is common in patients with ALL and it is associated with the inappropriate transcriptional silencing of genes implicated in the pathogenesis and prognosis of the disease [10], [11], [12], [13], [14]. This alteration represents an alternative mechanism to gene suppressor mutations and deletions and may affect indirectly the function of these genes in a similar fashion as mutations of *TP53* [7].

In the current study by analyzing 48 ALL patients samples using methylation bead arrays and subsequent validation of the results in a second series of 200 patients we demonstrate that aberrant DNA methylation is a common epigenetic alteration in ALL. Some of the genes found inappropriately methylated are involved in the p53 pathway suggesting that despite the lack of activating mutations of *TP53* in ALL, there is an abnormal function of p53 mediated by epigenetic mechanisms. Hypermethylation of genes implicated in the *TP53* pathway is a poor independent prognostic factor in patients with ALL and the use of compounds that can directly or indirectly activate this pathway opens new therapeutic strategies for patients with ALL.

## Results

### ALL displays marked aberrant promoter DNA methylation

**Figure 1:**
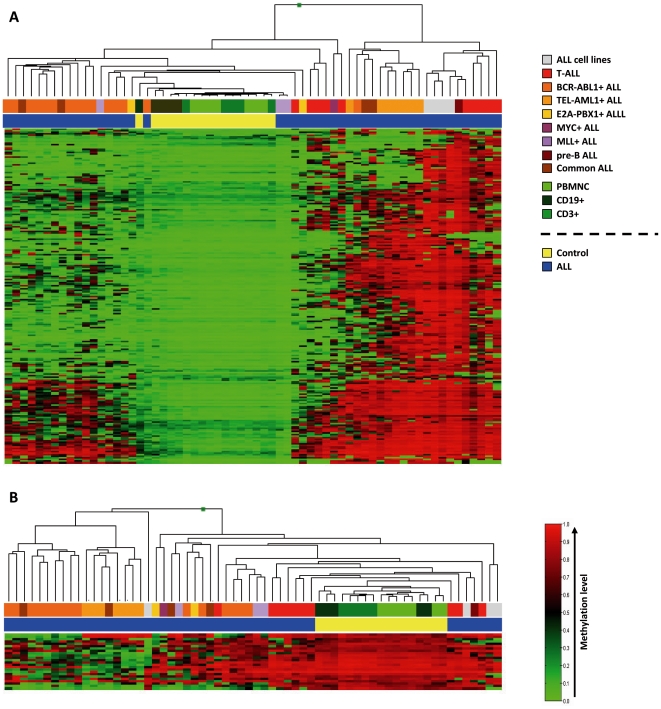
Hierarchical cluster analysis based on abnormally methylated genes in more than 10% of ALL samples. **A**. Hypermethylated genes in more that 10% of ALL samples. **B**. Hypomethylated genes in more than 10% of ALL samples. The top bar beneath the dendrogram refers to the subtypes of ALL and specific controls from healthy donors, while the lower bar indicates ALL or control. Subtypes of leukemia or controls are color coded (ALL: Acute Lymphoblastic Leukemia; PBMNC: Peripheral Blood mononuclear cells from healthy donors, CD19^+^ cells from PB and CD3^+^ cells from PB). Red: methylated; Green: non-methylated.

In the initial methylation profiling analysis using GoldenGate® Methylation Cancer Panel I array we included 17 samples from healthy donors (7 PBMNC, 5 CD19-positive cells from PBMNC and 5 CD3-positive cells from PBMNC), 48 ALL patient samples (17 BCR-ABL1^+^, 7 TEL-AML1^+^, 3 MLL^+^, 3 MYC^+^, 2 E2A-PBX1^+^, 4 common ALL, 1 pro-B ALL, 1 pre-B ALL and 10 T-cell ALL) and 4 ALL cell lines. Additionally, 5 of the 48 ALL samples showed more than 5% CpGs with detection p values >0.01 (Figure S1) and therefore were removed from further analyses. ALL samples (43 patients and 4 cell lines) had higher levels of de novo DNA methylation than control samples (n = 17) and clustered separately from them (Figure S2). The analysis of differentially methylated genes (DMA) is shown in Table S5. A total of 460 CpGs (299 genes) were hypermethylated in at least one ALL sample and 216 CpGs (154 genes) were hypermethylated in more than 10% of ALL samples (Figure 1A). Eighty eight CpGs (75 genes) were hypomethylated in at least one ALL and 24 CpGs from 22 genes were hypomethylated in more than 10% of ALLs (Figure 1B).

Previous reports have shown that genes acquiring the novo hypermethylation in cancer are targeted by the polycomb repressor complex 2 (PRC2) in embryonic stem cells (ESCs), indicating that such polycomb target genes seem to be predisposed for cancer-specific hypermethylation. This phenomenon has been shown for several tumors such as breast or colon cancer [15], [16]. In order to determine whether this also occurs in ALL, we analyzed the level of enrichment for PRC2 targets in ESC [17] in different ALL methylation groups. Interestingly, 47.2% of the genes aberrantly methylated in more than 10% of the ALL samples were PRC2 targets in ESC (only 21% of the genes included in the array were PRC2 targets; p<0.001). In contrast, only 9.1% of the hypomethylated genes were PRC2 targets in ESC. These findings suggest that indeed ALL-specific hypermethylation affects preferentially genes marked for repression by PRC2 in ESCs.

As hypermethylation frequently targets genes with dense CpG islands, we also studied the CpG content of differentially methylated groups in ALL samples. Among the 759 cancer related genes included in the array 58.8% corresponded to high CpG promoters (HCP), 14% to intermediate CpG (ICP) and 27.2% to low CpG (LCP) [18]. Hypomethylated genes were enriched in LCP (59.1%) (p = 0.03 in comparison with the whole group) while an enrichment of HCP promoters (78.2%) was observed among hypermethylated genes (p<0.001). These results indicate that ALL hypermethylation occurs in dense CpG islands while hypomethylation is more frequently observed in genes with low-density CpG dinucleotides.

The results from the microarrays were confirmed by the analysis of 12 genes included in the array by standard MSP and 3 out of 12 genes by pyrosequencing (Figure S3 and S4). The methylation status of these genes was analyzed in two ALL derived cell lines (TOM-1 and NALM-20), human male genomic DNA universally methylated for all genes and peripheral blood sample of healthy donor and compare with the equivalent samples in the array. By MSP, 8 out of 12 genes (*CDH1, CDH13, DBC1, SFRP1, SYK, TAL1, TP73* and *WNT5A*) were methylated in both ALL derived cell lines, 3 were unmethylated (*CDKN1A, FHIT* and *RASSF1A*) and 1 was methylated in TOM-1 cell line and unmethylated in NALM-20 cell line (*MGMT*) (Figure S2 and S3) [10], [19], [20], [21], [22]. By pyrosequencing, 2 out 3 genes (SYK and TAL1) were methylated in both ALL derived cell lines and 1 was methylated in TOM-1 cell line and unmethylated in NALM-20 cell line (*MGMT*) (Figure S3). The results obtained from the array and the individual analyses by MSP or pyrosequencing were highly concordant (Median of Beta values for genes unmethylated by MSP was 0.04 whereas for genes methylated by MSP was 0.84). These results indicate, as previously shown [23] that individual CpGs from methylation arrays can be taken as surrogate markers for the methylation status of the respective promoter regions in ALL.

### *TP53* pathway deregulation in ALL is associated with abnormal DNA hypermethylation of gene promoters implicated in p53 function

**Figure 2:**
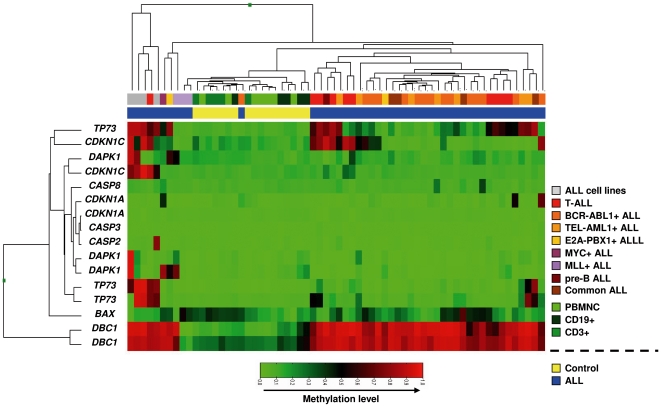
Hierarchical cluster analysis of *TP53* pathway genes included in the beadarray. The top bar beneath the dendrogram refers to the subtypes of ALL and specific controls from healthy donors, while the lower bar indicates ALL or control. Different subtypes of leukemia or controls are color coded (ALL: Acute Lymphoblastic Leukemia; PBMNC: Peripheral Blood mononuclear cells from healthy donors, CD19^+^ cells from PB and CD3^+^ cells from PB). Red: methylated; Green: non-methylated.

Some of the genes identified as hypermethylated by the arrays have already been described to be epigenetically regulated in ALL [10], [19], [20], [21], [22]. Four of these hypermethylated genes (*DBC1, TP73, DAPK1* and *CDKN1C*) have been implicated in the *TP53* pathway in ALL. Due to the fact that deregulation of the *TP53* pathway is a hallmark of cancer while function of p53 is abnormal in ALL despite the rare occurrence of *TP53* mutations in ALL [7], the abnormal hypermethylation of these genes prompted us to perform a new unsupervised analysis with all the genes implicated in the *TP53* pathway included in the Illumina array. The hierarchical clustering including all samples demonstrated that *TP53* pathway genes are aberrantly methylated in ALL samples and that this alteration accurately differentiates ALL from control samples (Figure 2).

**Figure 3:**
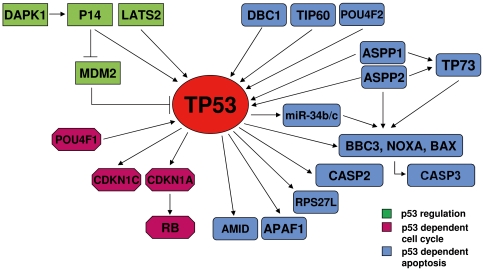
Outline of the genes involved in the *TP53* pathway.

**Figure 4:**
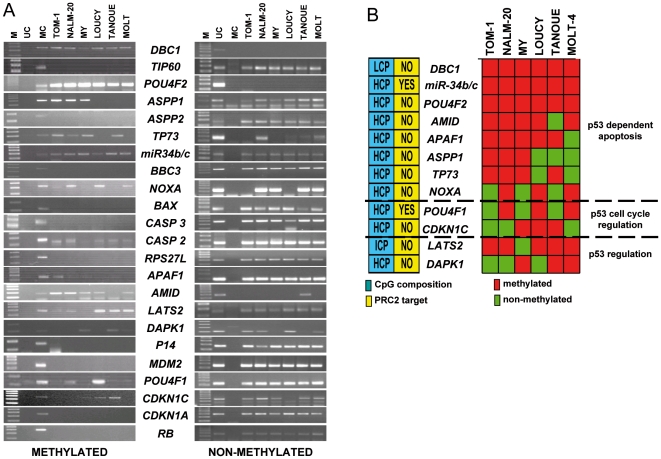
*TP53* pathway genes are methylated in ALL cell lines. **A.** MSP analysis of the methylated and un-methylated sequences of genes implicated in *TP53* pathway in ALL derived cell lines. UC: un-methylated control (DNA of peripheral blood from a healthy donor). MC: universally methylated control, M: 100 bp molecular marker. **B.** Graphic representation of the results of methylation of genes of *TP53* pathway and its involvement in *TP53* dependent apoptosis, cell cycle or regulation of *TP53*. Information regarding CpG composition and whether these genes are target of PCR2 in ESC is shown. Red: methylated, Green: unmethylated.

To further analyze the hypothesis that the *TP53* pathway might be inactivated or blocked in ALL independently of *TP53* mutations, we extended the analysis of DNA methylation by MSP to a total of 24 genes implicated in the *TP53* pathway using six derived ALL cell lines (Figure 3). These genes are implicated in p53 regulation and p53 dependent cell cycle and apoptosis while all of them have a typical CpG island. Interestingly 13 of the 24 genes were aberrantly hypermethylated in ALL derived cell lines (Figure 4A), of which 84.6% (11/13) were HCP, 7.7% (1/13) ICP and 7.7% (1/13) LCP. Unlike the whole group of genes differentially methylated in ALL, only 3 of the 13 genes (23.07%) of the *TP53* pathway were PRC2 targets in ESC (Figure 4B). Nine of the 13 methylated genes were involved in the regulation of p53 dependent apoptosis (*DBC1, hsa-miR-34b, hsa-miR-34c, POU4F2, AMID, APAF1, ASPP1, TP73* and *NOXA*), 2 of them in p53 dependent cell cycle regulation (*POU4F1* and *CDKN1C*) and the other two in p53 regulation (*LATS2* and *DAPK1*) (Figure 4B). While we have previously demonstrated most of these genes to be regulated by hypermethylation in ALL [11], [19], that was not the case for *AMID, POU4F1, POU4F2 and hsa-miR-34b/c*. Expression of these five genes was decreased in hypermethylated ALL cell lines and treatment *in vitro* with 4 µM of 5-aza-2-deoxycytidine (added one time only) during 4 days, induced a demethylation and upregulation of gene expression (Figure S5). These results indicate that DNA methylation of p53 dependent genes is an important mechanism associated with abnormal function of the *TP53* pathway in ALL.

### Activation of *TP53* pathway induces cell apoptosis in ALL

**Figure 5:**
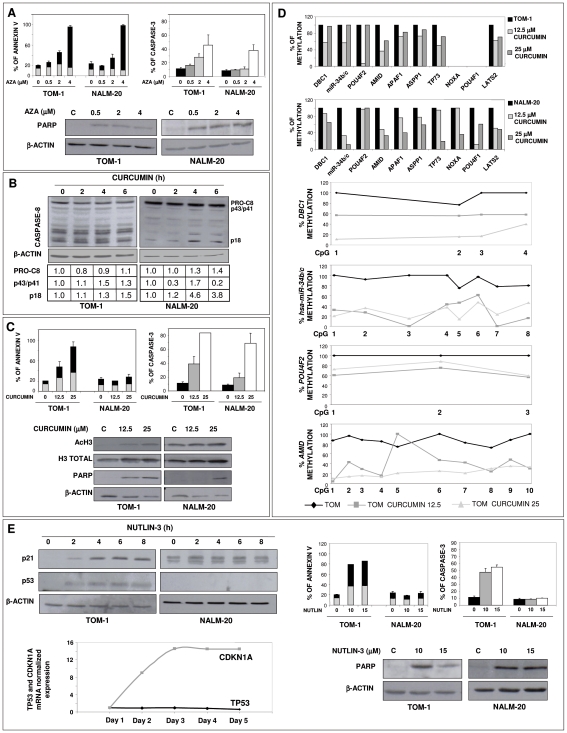
Activation of *TP53* function induces cell apoptosis in ALL. TOM-1 and NALM-20 cells were treated with 5-aza-2-deoxycitidine, Curcumin and Nutlin-3. **A**. Apoptosis induced by 5-aza-2-deoxycitidine treatment: Annexin V analysis by flow citometry (grey: early apoptotic; black: late apoptotic/death cells), Caspase-3 analysis by flow citometry and Western blot analysis of the 85-kDa fragment of PARP. **B–D.** Curcumin treatment; Western blot analysis and quantification of Caspase 8. PRO-C8: full length caspase-8 fragment; p43/p41: cleaved intermediate p43/p41 fragment of caspase-8 and p18: caspase-8 active fragment p18. Numbers at the bottom of the figure represent the quantification of these three fragments of caspase-8 (B); Activation of apoptosis measure by Annexin V (grey: early apoptotic; black: late apoptotic/death cells) and Caspase-3 (FACS) and by the detection of the 85-kDa fragment of PARP (C). Upregulation in the amount of acetylated histone 3 by Western blot is shown. Total histone 3 and β-actin are loading controls (C); qRT-MSP and pyrosequencing of hypermethylated genes implicated in *TP53* pathway before and after treatment with Curcumin (D). **E.** Western blot analysis of p21 and p53, levels of CDKN1A and TP53 mRNA by Q-RT-PCR and detection of apoptosis by Annexin V (grey: early apoptotic; black: late apoptotic/death cells), Caspase-3 and detection of the 85-kDa fragment of PARP by western blot after treatment with Nutlin-3. β-actin was used as a loading control in all cases. **A,C** and **E**, the mean ± SD of at least 3 different experiments is shown. **A-E**, a representative example of at least 3 different experiments is shown.

In order to prove that abnormal hypermethylation of genes implicated in the *TP53* pathway could be responsible at least in part for the resistance to apoptosis in ALL, TOM-1 and NALM-20 cells were treated with either a demethylating agent (5-aza-2′-deoxycytidine), a caspase-8 activator (Curcumin) that induces apoptosis downstream and independently of p53 and Nutlin-3, an inhibitor of MDM2 and thus an activator of the p53 pathway. We have previously demonstrated that TOM-1 shows no *TP53* mutation or deletion while NALM-20 shows a deletion of one *TP53* allele [11]. Treatment of ALL cells with 5-aza-2′-deoxycytidine induced a non-specific demethylation and upregulation of expression of genes implicated in *TP53* pathway (Figure S4), as well as an increase in apoptosis of TOM-1 and NALM-20 cells as measured by Annexin-V, caspase-3 or PARP expression (Figure 5A).

When ALL cells were treated with the caspase 8 activator Curcumin, pro caspase 8 was activated leading to an increase in apoptosis in both TOM-1 and NALM-20 cells (Figure 5B, C). Interestingly, beside an increase in apoptosis, treatment with Curcumin induced an increase in acethylation of histone 3 and demethylation of *TP53* pathway genes (Figure 5C, D) suggesting a role as an epigenetic drug for this compound. Finally, to examine if constant activation of *TP53* could induce a beneficial effect, ALL cells were treated with the MDM2 inhibitor Nutlin-3. Nutlin-3 induced apoptosis of TOM-1 cells which was associated with p53 accumulation, upregulation of *CDKN1A* transcription and an increase in p21 protein as a reflection of p53 dependent induction of apoptosis. In contrast, treatment of NALM-20 cells with Nutlin-3 did not induced p53 accumulation or increase of apoptosis, probably related to the fact that one allele of *TP53* is deleted in NALM-20 (Figure 5E).

### Hypermethylation of *TP53* pathway genes predicts clinical outcome in ALL patients

**Figure 6:**
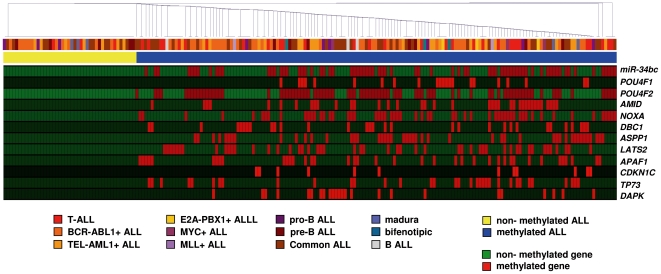
Hierarchical cluster analysis based on the methylation status of 13 genes of *TP53* pathway in ALL samples. Different subtypes of leukemia are coded by a specific colour. Yellow: ALL samples without methylation, Blue: ALL samples with a least one methylated gene.

We next analyzed the methylation status of the 13 genes inappropriately methylated in ALL cell lines in a group of 200 samples from ALL patients at diagnosis. The methylation frequencies (in descending order) for each of the 13 genes were: 41% for *POU4F2*, 36% for *hsa-miR-34bc*, 28% for *NOXA*, 24% for *LATS2*, 24% for *ASSP1*, 20% for *APAF1*, 19% for *AMID*, 17% for *TP73*, 16% for *DBC1*, 12% for *DAPK1*, 11% for *POU4F1* and 6% for *CDKN1C*. No methylated genes were found in 43 of 200 patients (non-methylated group, 22%) whereas most ALLs [157 (78%) of 200] had at least one gene methylated (methylated group). Based in the presence of gene promoter methylation of the 13 genes, the analysis performed using GENESIS (version 1.7.5) clustered separately patients with non-methylated genes and patients with at least one gene methylated (Figure 6). Among patients in the methylated group, 61% presented two or more genes methylated while none of the patients were found to have methylation of more than 9 genes. These results indicate that DNA methylation of *TP53* pathway genes is frequent in ALL and usually involves several genes. Similar to ALL cell lines, genes involved in p53 dependent apoptosis were more frequently methylated (70% of the patients) than p53 cell cycle dependent genes (15%) or p53 regulatory genes (32%). Methylation was more frequently observed in adult ALL than in children ALL (85% vs 70%, P = 0.015). Furthermore, the analysis clustered patients in only 2 groups, one including patients with none of the 13 genes methylated and another one including 1 or more methylated genes, reinforcing the hypothesis that the presence of a single methylated gene, more than the number of methylated genes is what confers a poor prognosis in ALL.

CR rates for patients in the non-methylated and methylated groups were 91% (39/43) and 84% (133/157), respectively (*P* = 0.3) with an overall CR rate of 86%. Patients in the non-methylated group had a lower relapse rate than patients in the methylated group (9/43, 21% vs 73/157, 46%, *P* = 0.001). Mortality rate was also lower for the non-methylated group compared with the methylated group (8/43, 19% versus 88/157, 56%, *P*<0.001). Similar results were observed when adults and children were analyzed separately: relapse rate was 18% for children in the non-methylated group versus 37% for the methylated group, *P* = 0.08; mortality rate was 4% for children in the non-methylated group versus 27% for methylated group, *P* = 0.01); relapse rate and mortality in adults were 31% for non-methylated group versus 65% for methylated group, *P* = 0.03 and 44% for non-methylated group versus 76% for methylated group, *P* = 0.01 respectively.

**Figure 7:**
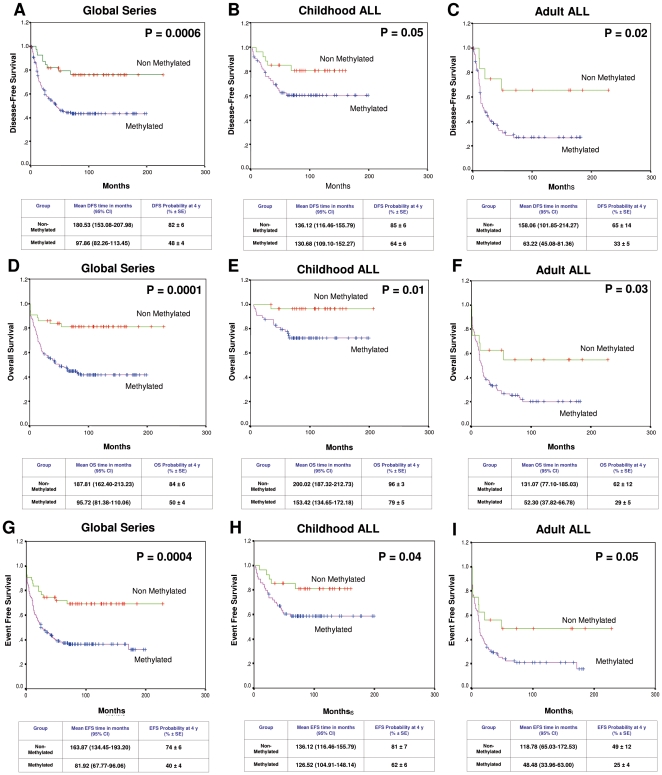
Survival curves of Kaplan-Meier. DFS curves for all patients enrolled in this study (panel A), childhood ALL (B) and adult ALL (C) according to the methylation profile. OS curves for all patients enrolled in this study (D), childhood ALL (E) and adult ALL (F) according to the methylation profile. EFS curves for all patients enrolled in this study (G), childhood ALL (H) and adult ALL (I) according to the methylation profile.

Survival data were available on the whole cohort, with a median follow-up until censoring from any cause of 50.9 months (95% CI: 35.9–66 months). Median follow-up times to relapse and death were 24 months (95% CI: 12.7–35.3 months) and 13.2 months (95% CI: 10.9–15.4 months), respectively. Disease free survival at 16 years was 76% and 43% for non-methylated and methylated group (*P* = 0.0006) (Figure 7A). Among children, the 14-year DFS was 81% for non-methylated group and 61% for methylated group (*P* = 0.05) (Figure 7B) while adult ALL patients had a 15-year DFS of 66% for non-methylated group and 27% for methylated group (*P* = 0.02) (Figure 7C). The actuarial overall survival (OS) at 16 years calculated for all leukemic patients was 81% for non-methylated patients and 41% for methylated patients (*P* = 0.0001) (Figure 7D). Significant differences were observed in the actuarial OS among non-methylated and methylated groups in the separate analyses of children (96% vs 72%, *P* = 0.01) (Figure 7E) and adults (55% vs 20%, *P* = 0.03) (Figure 7F). Event free survival at 15 years was 69% and 32% for non-methylated and methylated group (*P* = 0.0004) (Figure 7G). Among children, the 14-year EFS was 81% for non-methylated group and 58% for methylated group (*P* = 0.04) (Figure 7H) while adult ALL patients had a 15-year EFS of 49% for non-methylated group and 16% for methylated group (*P* = 0.05) (Figure 7I). The multivariate analysis of potential prognostic factors demonstrated that hypermethylation was an independent prognostic factor for DFS (*P* = 0.006) (Table S6), OS (*P* = 0.005) (Table S7) and EFS (*P* = 0.01) (Table S8).

## Discussion

The number of genes regulated by epigenetic mechanisms including DNA hypermethylation and histone modifications in patients with ALL has raised significantly in the last few years, indicating that epigenetic changes play a significant role in the pathogenesis and prognosis of the disease [10], [12], [14], [24], [25], [26], [27], [28]. Our current results not only further increase the number of genes described as aberrantly methylated in ALL but more importantly indicate that one of the pathways most frequently deregulated in different cancers such as *TP53* is altered in ALL not due to genetic events but rather to the abnormal hypermethylation of many genes involved in p53 induced apopotosis, cell cycle and p53 regulation. The higher frequency of hypermethylation among gene promoters with HCP and a higher percentage of hypomethylated genes among LCP promoters supports the model recently described in which de novo methylation in cancer mainly affects genes with dense CpG islands [23].

Recent studies have described genome wide methylation in ALL using different approaches such as differential methylation hybridization [24], CGP-island arrays [25], [26], [27], MCA/human proximal promoter DNA microarray [14] or HELP assays [28]. These methods vary in terms of number and type of genomic regions detected, quantification accuracy and resolution. The CpG-specific array used in the present study is highly quantitative and shows a single-base resolution. However, as compared to other arrays with higher coverage, it can only detect changes in 800 cancer-related genes and 1 to 3 CpGs are studied per gene. Even considering this lower coverage, we could detect a large number of genes (n = 154) hypermethylated in more than 10% of the ALL samples. Among the studies quoted above, those including more than 400 ALL patients have shown that certain genetic subtypes maybe distinguished based on their methylation profile [25] while others have focused on specific subgroups of ALL such as MLL leukemia, demonstrating that hypermethylation is associated with a worse prognosis [24]. In our study there was no specific methylation profiles associated with the cytogenetic group probably due to the limited number of patients per group.

Genetic abnormalities are in part responsible for the development of the disease; however, as these may arise in utero, there is long latency before overt ALL, showing that additional changes are needed [29]. The potential to contribute to leukemia development varies among different translocations so while BCR-ABL1 is strong leukemogenesis inducer, other translocations such as *TEL-AML1* requires more events. Gene deregulation through hypermethylation may contribute to the transformation of the hematopoietic cells. In line with this hypothesis, BCR-ABL1+ ALL samples contained a smaller number of hypermethylated genes than TEL-AML1 ALL or T-ALL samples which could be related to the higher leukemogenesis potential of this oncogene. The results are also consistent with previous observations from our group in patients with Ph^+^ CML in which DNA hypermethylation is an infrequent event [23], [30] with DNA hypomethylation being a more representative epigenetic alteration [31].

Recent studies have demonstrated that genes inappropriately hypermethylated in cancer are frequent targets of PRC2 in embryonic stem cells supporting the hypothesis that de novo methylation is pre-programmed by an established epigenetic system that predisposes tumor suppressor genes to DNA hypermethylation [15], [16]. This hypothesis is also supported by our results showing that almost half of the genes aberrantly methylated in more than 10% of the ALL samples were PRC2 targets in embryonic stem cells [17]. Whether the epigenetic alteration could be the initiating event that predisposes a precursor cell to transformation or alternatively the genetic changes (such as translocations) are the initial transformation event that predisposes the cells to further epigenetic changes is unclear. Experimental data supports that certain genetic alterations such as the PML-RAR fusion protein has the potential to form a stable complex with DNMT1 and DNMT3a that subsequently can induce gene promoter hypermethylation [32].

Frequent and simultaneous aberrant methylation of genes implicated in *TP53* pathway indicates that, although indirectly and against previous knowledge, *TP53* is altered in a high percentage of ALL patients, as it is the case in other types of cancer. Hypermethylation of these 13 genes have been described in independent studies in other types of tumors such as *DBC1* in lymphomas and lung cancer [33], *LATS2* in head and neck squamous cell carcinoma, astrocytoma or breast cancers [34], *ASPP1* in hepatocellular carcinoma [35], *DAPK1* in Multiple Myeloma [36] and chronic lymphocytic leukemia [37], *APAF1* in acute myeloblastic leukaemia [38] and gastric cancer [39], *TP73* in cervical carcinomas [40], *CDKN1C* in diffuse large B cell lymphoma [41] and pancreatic ductal neoplasms [42] and finally, *hsa-miR-34b/c* in ovarian and colorectal cancers [43]. The use of the bead-array based technology in patients with lymphoma [23], colorectal cancer [44] and glioblastoma multiforme [45] have shown that similar to what we observed in ALL, a frequent and simultaneous methylation of some of these genes is a frequent event in other tumors indicating that in addition to classical mutations and deletions of *TP53*, we should take into account indirect inactivation of *TP53* in other types of human cancers.

An important clinical implication from our results is that we establish *TP53* pathway as an attractive therapeutic target in ALL. The development of drugs that target the most frequent alterations in human cancer such as the universal alteration of the *TP53* pathway has been a major quest in cancer research [46]. Currently, two different approaches are being used: a molecule that directly activate p53 by blocking protein-protein interactions and compounds that trigger indirectly the stimulation of *TP53* system [46] such as Nutlin-3, a small molecule antagonist of MDM-2 that disrupts the MDM2-p53 interaction resulting in p53 stabilization and activation of p53 function [47]. Treatment of ALL cells with Nutlin-3 induced cell apoptosis in TOM-1 which was consistent with stabilization of the p53 protein (Figure 5) but not in NALM-20 cells, probably because these cells have the deleted one allele of *TP53*.

Curcumin is the natural polyphenolic antioxidant and most active component of curry spice. It has been used for over 4000 years in the traditional Indian, Chinese or Arabic medicine and has demonstrated a low toxicity in humans even at high doses showing a remarkable anti-inflammatory, antioxidant, pro-apoptotic and anti-tumor properties [48]. We used Curcumin in our study based on its potential to directly induce apoptosis by activation of caspases thus preventing the need for functional p53. Interestingly, Curcumin in addition to activation of caspase 8, was able to induce epigenetic mechanisms such as acetylation of histone 3 or DNA demethylation suggesting a role for these mechanisms in apoptosis induced by Curcumin in ALL cells, making this compound an attractive candidate for further studies.

In summary, we show that aberrant DNA methylation is common and occurs simultaneously in several genes in ALL cells, directly affecting an essential pathway implicated in tumorigenesis such as the tumor suppressor gene *TP53*. Hypermethylation of genes implicated in *TP53* pathway is an independent prognostic factor for DFS, OS and EFS in patients with ALL. The use of compounds that can directly or indirectly activate this pathway such as 5-aza-2-deoxycitidine, Curcumin or Nutlin-3 opens new therapeutic strategies for patients with ALL.

## Materials and Methods

### Human samples and cell lines

The following ALL-derived cell lines (TOM-1, NALM-20, MY, LOUCY, TANOUE and MOLT-4) were used for *in vitro* studies. Cell lines were maintained in culture in RPMI 1640 medium supplemented with 10% fetal bovine serum with 1% penicillin-streptomicin and 2% hepes at 37°C in a humid atmosphere containing 5% CO2. Bone marrow mononuclear cells were obtained at diagnosis from patients with ALL after informed consent was obtained from the patient or the patient's guardians, in accordance with the Declaration of Helsinki. This study was approved by the Research Ethics Committee at the University of Navarra. Some of the samples were obtained more than 15 years ago. In these cases, consent was verbal and as such was accepted by the Research Ethics Committee. Samples from recent patients were obtained with written informed consent. Samples were divided in 2 groups for *methylation arrays* (48 patients) and *validation analysis* (200 patients). Diagnosis was established according to standard morphologic, cytochemical and immunophenotypic criteria and all patients were enrolled in successive multicenter studies of the “*Programa para el estudio y tratamiento de las hemopatias malignas*” (PETHEMA) Spanish study group. Samples used in the arrays included BCR-ABL1^+^ ALL t(9;22) (n = 17), TEL-AML1^+^ t(12;21) (n = 7), MLL^+^ (n = 3), MYC^+^ (n = 3), E2A-PBX1^+^ t(1;19) (n = 2), common ALL (n = 4), pro-B ALL (n = 1), pre-B ALL (n = 1) and T-cell ALL (n = 10). In the validation group, 200 samples (113 male and 87 female) from patients diagnosed with *de novo* ALL between January 1990 and May 2007 were included. Median age at diagnosis was 17 years (range 0.5–82 years) with 91 children (median age 5, range 0.5–14 years) and 109 adult patients (median age 36; range 15–82 years). Patients were risk-stratified according to the therapeutic protocol used, which was always based on recognized prognostic features (including cytogenetics) and were entered in ALL protocols of the PETHEMA Spanish study group. For statistical analyses, children were also grouped according to the National Cancer Institute (NCI) risk-classification criteria [49]. The specific PETHEMA ALL treatment protocols in which these patients entered included ALL-89 (between 1990–1993; n = 48) and ALL-93 (between 1993–2007; n = 152). The design and clinical results of these studies have been previously reported [50], [51], [52]. Eighty-two patients relapsed. Forty-two patients received stem cell transplantation (SCT, 8 autologous, 34 allogeneic) in the first (n = 14) or second complete remission (CR) (n = 20). There are 104 patients currently alive. Clinical characteristics of the patients are listed in Table S1.

For methylation arrays, samples from 17 healthy donors obtained from male and female Caucasian European volunteers between 25–40 years old, were included. (Peripheral Blood Mononuclear Cells n = 7; CD19-positive cells from PBMNC n = 5 and CD3-positive cells from PBMNC n = 5). CD3^+^ and CD19^+^ cells were enriched by Magnetic Cell Sorting using CD3 or CD19 human microbeads, respectively (Miltenyi Biotec, Cologne, Germany) and the AutoMACS selection device. In all cases cell purity was over 90%.

### DNA methylation-specific array and data analysis

For genome-wide DNA methylation analyses, we used the GoldenGate® Methylation Cancer Panel I (Illumina, Inc.), which contains 1505 CpG sites selected from 807 cancer-related genes. The experimental protocol, scanning, image processing and data extraction was carried out as previously described according to the instructions of the manufacturer [23], [53], [54]. Before analyzing the methylation data (so called beta values, which range from 0 for unmethylated and 1 for completely methylated), we excluded possible sources of biological and technical biases that could alter the results. As one copy of chromosome X is methylated in women, we excluded all 84 CpGs on chromosome X to avoid a gender-specific bias. Additionally, we evaluated the detection probabilities (comparing signal intensities against background noise) for all CpGs and excluded those CpGs with values of P>0.01 in more than 15% of cases (Figure S1A). From a total of 1505 CpGs included in the array, 60 CpGs showed bad detection p values (i.e. above 0.01) in more than 15% of cases and consequently were eliminated from the study. Additionally, samples with P>0.01 in more than 5% of the CpGs were removed from the analyses (Figure S1B). Using these criteria, 1367 autosomal CpGs from 759 genes entered further statistical analyses, and 5 ALL samples were removed because of their poor quality.

Unsupervised hierarchical clustering was performed using the Cluster Analysis tool of the BeadStudio software (version 3.2). As DNA methylation usually follows a bimodal distribution (a gene is either methylated or unmethylated), a differential methylation analysis was performed by categorizing the methylation data in controls and cases. Considering that some genes might be clearly differentially methylated in a small subset of ALL cases, a CpG was classified as hypermethylated in ALL when the mean in the controls was homogeneously below 0.25 (and none of them was above 0.5) and individual beta values were greater than 0.75 in at least 10% of the ALL samples. Conversely, a CpG was considered hypomethylated in ALL when the mean in the controls was homogeneously above 0.75 (and none of them was below 0.5) and individual beta values were smaller than 0.25 in at least 10% of the ALL samples. CpGs that could not be assigned to any of these groups, v.g. CpGs methylated or unmethylated in cases and controls, as well as CpGs with mean methylation values between 0.25 and 0.75 (partially methylated) in the control samples, were not considered for further analyses.

### Methylation-Specific PCR (MSP)

DNA of cell lines was extracted using QIAmp DNA Mini Kit (Qiagen, Hilden, Germany). MSP was used to analyze the methylation status of the gene promoter region as previously described [10], [55]. This technique is based on a bisulfite treatment of the DNA that modifies unmethylated but not methylated cytosines into uracils. 1 µg of genomic DNA was treated and modified using the CpGenomic DNA modification Kit (Millipore, Billerica, MA01821, USA). After bisulfite treatment of DNA, “hot start” PCR was performed for 35 cycles consisting of denaturation at 95°C for 1 min, annealing at T^a^ for 1 min, and extension at 72°C for 1 min, followed by a final 72°C for 10 min extension for all primer sets. Primer sequences of each gene for the unmethylated and methylated reactions are described in Table S2 and S3. The products were separated by electrophoresis on 1.8% agarose gel. Bone marrow and peripheral lymphocyte DNA from healthy donors were used as negative control for methylation-specific assays. Human male genomic DNA universally methylated for all genes (Intergen Company, Purchase, NY) was used as a positive control for methylated alleles. Water blanks were included with each assay. The results were always confirmed by repeat MSP assays after an independently performed bisulfite treatment. Methylation status of *AMID, POU4F1, POU4F2, hsa-miR-34b* and *hsa-miR-34c* CpG islands after 5-aza-2'-deoxycytidine and *DBC1*, *hsa-miR-34b, hsa-miR-34c, POU4F2, AMID, APAF1, ASPP1, TP73, NOXA, POU4F1 and LATS2* after Curcumin treatment was performed by semiquantitative real time methylation-specific PCR (qRT-MSP) as previously reported [55].

To validate the DNA methylation data generated by BeadArray technology, methylation-specific-PCR (MSP) of 12 genes were performed as previously described [10], [19], [20], [21], [22], [56], [57], [58]. For *TAL1*-MSP, TAL1-MD (5'- GTATAGTTCGGTGGTGGGTATTC-3') and TAL1-MR (5'- CGCACCTAATCCTACTAAACGAC -3') primers were used for the methylated reaction and TAL1-UD (5'-TTGTATAGTTTGGTGGTGGGTATTT-3') and TAL1-UR (5'- CCCACACCTAATCCTACTAAACAAC -3') primers for the unmethylated reaction. PCR conditions for TAL1-MD/TAL1-MR and TAL1-UD/TAL1-UR primers were 94°C for 10 min, followed by 35 cycles at 94°C for 1 min, 60°C for 1 min and 72°C for 1 min. The final extension was at 72°C for 10 min in both cases. MSP products were separated on a 2% agarose gel, stained with ethidium bromide and visualized under UV light. Results were confirmed by repeating bisulfite treatment and MSP assays for all samples.

### DNA methylation analysis by Pyrosequencing

The PCR reactions for pyrosequencing were done with a biotynilated specific reverse primer and performed as follows. After bisulfite treatment of DNA, “hot start” PCR was performed with a denaturalization at 95°C for 10 minutes and for 35 cycles consisting of denaturation at 95°C for 1 min, annealing at the specific temperature for each gene for 1 min, and extension at 72°C for 1 min, followed by a final 10 min extension for all primer sets. Primer sequences of each gene are described in Table S4. The resulting biotinylated PCR products were immobilized to streptaviding Sepharose High Performance beads (GE healthcare, Uppsala, Sweden) and processed to yield high quality ssDNA using the PyroMark Vacuum Prep Workstation (Biotage, Uppsala, Sweden), according to the manufactureŕs instructions. The pyrosequnecing reactions were performed using the Pyromark^TM^ ID (Biotage, Uppsala, Sweden) and sequence analysis was performed using the PyroQ-CpG analysis software (Biotage, Uppsala, Sweden). Bone marrow and peripheral lymphocyte DNA from healthy donors were used as negative control for methylation-specific assays. Human male genomic DNA universally methylated for all genes (Intergen Company, Purchase, NY) was used as a positive control for methylated alleles. Water blanks were included with each assay. The results were always confirmed by repeat pyrosequencing assays after an independently performed bisulfite treatment.

### Expression analyses by Q-RT-PCR

Total RNA was extracted from human bone marrow mononuclear cells or cell lines with Ultraspect (Biotecx, Houston, TX, USA) following the manufacturer's instructions. Reverse transcription and Q-RT-PCR were performed using specific primers for *AMID*, *POU4F1* and *POU4F2* (Table S9). Amplification of glyceraldehide-3-phosphate dehydrogenase (GAPD) transcript was performed to assess RNA integrity and as a reference gene using specific primers and TaqMan probe (Hs99999905_m1; Applied Biosystems, Foster City, CA).

Reverse transcription was performed on 1 µg of total RNA, after heating at 70°C for 5 min, with random hexamers as reaction primers. The reaction was carried out at 42°C for 45 min in the presence of 12 U Avian Myeloblastosis virus reverse transcriptase (Boehringer-Mannhein, Germany). Q-RT-PCR was performed in a rapid fluorescent thermal cycler with three-color fluorescence monitoring capability (LightCycler, Roche Molecular Biochemicals, Mannheim, Germany), using 1 µl of cDNA in 20 µl reaction volume with 0.4 µmol/l of each primer, and 2 µl of 10 LightCycler FastStar DNA Master SYBR Green I (Roche Molecular Biochemicals, Mannheim, Germany). The final Mg^2+^ concentration in the reaction mixture was adjusted to 3.5 mmol/l. The following program conditions were applied for Q-RT-PCR running: denaturation program, consisting in one cycle at 95°C for 8 min; amplification program, consisting in 45 cycles at 95°C for 5 s, 60°C for 10 s and 72°C for 15 s; melting program, one cycle at 95°C for 0 s, 40°C for 60 s and 90°C for 0 s; and cooling program, one cycle at 40°C for 60 s. The temperature transition rate was 20°C/s, except in the melting program, that was 0.4°C/s between 40 and 90°C. In order to reduce the variation between different assays and samples, a procedure based on the relative quantification of target genes versus their controls in relation to the reference gene was used. Calculations were automatically performed by LightCycler software (RealQuant, version 1.0, Roche Molecular Biochemicals, Mannheim, Germany). The normalized ratio was obtained from the next equation and expressed as a percentage of the control:
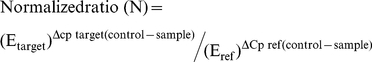



Efficiencies (E) for each gene were calculated from the slopes of crossover points (Cp) vs. cDNA concentration plot, according to the formula E = 10(-1/slope). ΔCp corresponds to the difference between control Cp and sample Cp, either for the target or for the reference genes. The selected control was a bone marrow specimen from a healthy donor and was considered as 100% expression. Occasionally, equal amounts of PCR products were separated on a 2% agarose gel, stained with ethidium bromide and visualized under UV light.

### Q-RT-PCR for *hsa-miR-34b/c*

Total RNA was extracted with Ultraspec (Biotecx, Houston, TX, USA) following the manufacturer's instructions. RNA concentration was quantified using NanoDrop Specthophotometer (NanoDrop Technologies, USA). 5 ng of total RNA was used to synthesize a specific cDNA of the *hsa-miR-34b* and *-34c* using a stem-loop miRNA-specific RT primer according to the TaqMan MicroRNA Assay protocol (Applied Biosystems, Foster City, CA). Reverse transcriptase reactions contained 1.5 µl of miRNA-specific stem-loop RT primer and 6 µl of Master Mix composed of 2 ng of RNA, 1x RT buffer, 0.25 mM of each dNTP, 3.33 U/µl of MultiScribe reverse transcriptase and 0.25 U/µl of RNase Inhibitor (Applied Biosystems, Foster City, CA, USA). The 7.5 µl reactions were incubated in a 2720 Thermal Cycler (Applied Biosystems, Foster City, CA) for 30 min at 16°C, 30 min at 42°C, 5 min at 85°C and then maintained at 4°C. Expression of the *hsa-miR-34b* and *-34c* was analyzed using specific primers and TaqMan probe (PD4373037 for *hsa-miR-34b* and PD4373036 for *hsa-miR-34c*) according to the TaqMan MicroRNA Assay protocol (Applied Biosystems, Foster City, CA). Quantitative real time-PCR (Q-RT-PCR) was performed in a 7300 Real Time System (Applied Biosystems, Foster City, CA), using 0.7 µl of RT product of *hsa-miR-34b* or *-34c* in a reaction volume of 10 µl with 1x TaqMan Universal PCR master mix and 1 µl of primers and probe mix according to the TaqMan MicroRNA Assay protocol (PE Applied Biosystems, Foster City, CA). The reactions were incubated at 95°C for 10 min, followed by 45 cycles of 95°C for 15 s and 60° for 30 s. The Ct data was determined using default threshold settings. The threshold cycle (Ct) is defined as the fractional cycle number at which the fluorescence passes the fixed threshold. Expression of *hsa-miR-34b* and *-34c* were normalized using the expression of *RNU6B* gene in each sample. For expression of *RNU6B* we used TaqMan RNU6B assay (PN: 4373381) (Applied Biosystems, Foster City, CA). The normalized ratios of *hsa-miR-34b* and *-34c* expression were calculated as described above. The selected control/calibrator was the bone marrow lymphocytes from a healthy donor that was considered as 100% of *hsa-miR-34b* or *-34c* expression.

### Cell treatments

TOM-1 and NALM-20 ALL-derived cell lines were treated with the demethylating agents 5-aza-2'-deoxycytidine (diluted in 1∶1 water∶acetyc) at 0.5, 2 and 4 µM (Sigma Aldrich, Steinheim, Germany); the caspase 8 activator Curcumin (diluted in DMSO) at 12.5 and 25 µM (Sigma Aldrich, Steinheim, Germany) and with the MDM2 inhibitor Nutlin-3 (diluted in DMSO) at 10 and 15 µM (Sigma Aldrich, Steinheim, Germany).

### Western blotting

Cells pellets were resuspended in lysis buffer containing 1% Triton X-100, 50 mM Tris HCl (Ph = 8), 150 mM NaCl, 1 Mm NaVO_4_, 10 mM NaF and 1X complete (Roche, Mannheim, Germany) for 30 minutes at 4°C. After centrifugation at 13000 rpm for 15 minutes at 4°C the supernatant was collected as whole cell lysates. Protein concentration was determined with the BCA assay (Pierce Chemical, West Pico, Rockville, IL). Equal amounts of protein (15 µg/lane for nuclear and cytoplasmic extract and 50 µg/lane for total extracts) were separated by 10% sodium dodecyl sulfate-polyacrylamide gel electrophoresis (SDS-PAGE) and transferred onto a nitrocellulose membrane (Bio-Rad, Hercules, CA). The membranes were blocked for 1 hour with 0.1% Tropix in 0.01% Tween 20 PBS and incubated with a monoclonal antibody against PARP p85 (1∶1000 for 1 hour at room temperature (RT) (Promega, Madison, WI), p-Tyr* (1∶1000 for 1 hour at RT. 05-321, Upstate), ABL1 (1∶2000 for 3 hours at RT. sc-23, Santa Cruz Biotechnology), Caspase-8 (1∶2000, o/n at RT. 9746, Cell Signaling), p53 (1∶5000 for 2 hours at RT. P6874, Sigma, St. Louis, MO), p21 (1∶1000, o/n at 4°C. 556430, PharMingen), Histone 3 (1∶50000 for 2 hour at RT. 06-599. Millipore, Billerica, MA01821, USA) and acetyl Histone 3 (1∶20000, o/n at 4°C. 07-690. Millipore, Billerica, MA01821, USA). Antibody against β-actin (1∶4000 for 1 hour at RT. Sigma, St. Louis, MO) was used to confirm equal loading of nuclear, cytoplasmic, and total extracts, respectively. Secondary antibody coupled with alkaline phosphatase (1∶10000 at RT. Sigma, St. Louis, MO) were added for an additional 1 hour. Inmunoreactive bands were developed using a chemiluminescent substrate (CSPD, Tropix) and Hyperfilm ECL plus films (Amersham, Arlington, Heights, IL). An autoradiograph was obtained with exposure times from 1 to 10 minutes.

### Apoptosis analysis

The apoptosis was studied by Annexin V and Caspase-3 analysis by flow citometry and the detection of the 85-kDa fragment of PARP by western blot. Annexin V apoptosis analysis: Cell death and apoptosis were determined by staining cells with annexin-V-FITC and counterstaining with propidium iodide (PI) using the FITC Annexin V Apoptosis Detection Kit I (BD Pharmingen, San Diego, CA) following the manufactureŕs instructions. Analyses were performed 24 hours after treatment on FACscan (Becton –Dickinson, Heilderberg, Germany), using the CellQuest analysis software. Viable cells with intact membranes exclude PI, whereas the membranes of dead and damage cells are permeable to PI. The FITC Annexin V staining precedes the loss of membrane integrity which accompanies the latest stages of cell death resulting from either apoptotic or necrotic processes. Therefore, cells which were Annexin-V-FITC-negative and PI-negative were classified as living cells, while PI-positive cells were classified as necrotic. Annexin-V-FITC-positive but PI negative cells were classified as early apoptotic as they present membrane integrity. The mean percentage of cells expressing annexin V, PI or both is shown. Active Caspase-3 apoptosis analysis: The FITC-Conjugated Monoclonal Active Caspase-3 antibody apoptosis Kit (BD Pharmingen, San Diego, CA) was used following the manufactureŕs instructions. The mean percentage of cells expressing active caspase-3 is shown.

### Statistical analysis

Remission status was assessed after completion of induction chemotherapy. Complete remission (CR) was defined as follows: granulocyte count of at least 1×10^9^/l, platelet count of at least 100×10^9^/l, no PB blasts, BM cellularity of at least 20% with maturation of all cell lines and less than 5% blasts, no extramedullary leukemia. Primary therapy failure was defined as persistence of PB blasts or at least 25% blasts in BM after induction therapy. Relapse was defined as reappearance of PB blasts, more than 5% blasts in BM, or appearance of extramedullary manifestations after CR was achieved. Overall survival (OS) was measured from the day of diagnosis until death from any cause and was censored only for patients known to be alive at last contact. Disease-free survival (DFS) was measured from the day that CR was established until either first relapse or death without relapse, and it was censored only for patients who were alive without evidence of relapse at the last follow-up. Event-free survival (EFS) was defined as time from study entry until removal from study because of failure to achieve complete remission, first relapse, or death from any cause. All relapse and survival data were updated on May, 2009, and all follow-up data were censored at that point. Patients who underwent SCT were included but censored at the date of transplant.

For statistical purposes, ALL patients were classified into two different methylation groups: Non-methylated (no methylated genes) and methylated group (at least, one methylated gene). The rationale for grouping patients with any methylated gene (regardless of the number of methylated genes) in the same methylation group was derived after statistical analyses for OS and DFS according to the number of genes methylated (from 0 to 9; no patients showed methylation of more than nine genes). Results of these analyses showed that prognosis for non-methylated patients were better than patients with methylated genes. Moreover, prognosis of patients with 1 to 9 methylated genes was similar with no statistical differences (data not shown). Comparisons of molecular and clinical features across groups were performed using the 2 or a 2-sided Fisher exact test for categorical data and the nonparametric Mann-Whitney U test for continuous variables. A P value less than or equal to .05 (2-sided) was considered significant. Distributions of OS, DFS and EFS curves were estimated by the method of Kaplan and Meier, with 95% confidence intervals calculated by means of Greenwood's formula and with the log-rank comparing differences between survival curves. Hazard ratios comparing DFS, EFS, and OS between subgroups were calculated using univariate Cox models. Multivariate Cox regression modeling was done for DFS, EFS and OS using a forward-selection stepwise modeling process (with a forward selection method with entry probability of *P* = .01. using Wald CIs and with stepwise removal of non-significant factors); the difference in the log likelihood (−2×log likelihood) was used, along with an adjustment for phase of trial (PETHEMA ALL89 vs PETHEMA ALL93). Factors were entered as categorical values as far as possible to keep the computations simple, although categorization inevitably results in some loss of information. All variables in the model were linear and conformed to the proportional hazards assumption. The following variables were considered in the model: age (≤15 vs. >15 years), methylation profile (negative vs. positive), WBC (≤50×10^9^/l vs. >50×10^9^/l, BCR-ABL (negative versus positive), cell immunophenotype (B versus T) and PETHEMA risk groups (high versus others). For children we also included TEL-AML1 (positive versus Negative) and NCI risk groups (high versus others).

## Supporting Information

Figure S1 **Definition of methylation analysis thresholds based on the distribution of bad detection p values (p>0.01).** A) Threshold definition per CpG. A total of 1505 CpGs were studied with the bead array. The threshold of 15% was set based on visual inspection of this distribution (i.e. the estimated inflexion point). Based on this threshold, a total of 60 CpGs (the right peak of the plot) showed bad p values in 15% or more of the cases. B) Threshold definition per case. A total of 69 hybridizations were performed including ALLs and controls. As shown in the figure, low numbers of bad p values (ranging from 1 to 5%) are seen in the first 64 hybridizations. However, the 5 cases showed a clearly higher percentage of bad p values, Based on this distribution, we set a threshold for case selection of 5% (i.e. a case is selected for further statistical analyses if 95% or more CpGs show good p values).
(TIF)

Figure S2 **Hierarchical clustering analysis of DNA methylation data.** Dendrogram of hierarchical cluster analysis based on the methylation status of CpG regions from ALL and control samples. The top bar beneath the dendrogram refers to the subtypes of ALL and specific controls from healthy donors, while the lower bar indicates ALL or control. Subtypes of leukemia or controls are color coded (ALL: Acute Lymphoblastic Leukemia; PBMNC: Peripheral Blood mononuclear cells from healthy donors, CD19^+^ cells from PB and CD3^+^ cells from PB). Red: methylated; Green: non-methylated.
(TIF)

Figure S3 **MSP melting curve analysis of the methylated sequences.** The presence of specific methylated products is shown by the melting curves obtained by means of a LightCycler 2.0 PCR real time device. Green: positive methylated control; pink: peripheral blood lymphocytes from healthy donors; yellow: blank control (water); red: TOM1 cell line; blue: NALM-20 cell line.
(TIF)

Figure S4 **Comparison results obtained by Illumina Beadarray, Methylation Specific PCR (MSP) and pyrosequencing.** **A.** Bead array methylation results. **B.** MSP results in same sample used in the bead array. Because several CpG for each gene on the array have been analyzed (A) and we have only one result of MSP (B), some of the MSP rows have been duplicated/triplicated to equal the number of CpGs. PB: Peripheral Blood of healthy donor, MC: human male genomic DNA universally methylated for all genes, Red: methylated, Green: non methylated. **C.** Pyrosequencing results of analyzed CpG loci on the array, corresponding to *MGMT*, *SYK* and *TAL1* genes. The values are expressed as percentage of methylation.
(TIF)

Figure S5 **DNA methylation and expression analysis of** ***hsa-miR-34b/c*** **,** ***POU4F1*** **,** ***POU4F2*** **and** ***AMID*** **in ALL cell lines.** **A.** Q-MSP analysis of *hsa-miR-34b/c, POU4F1, POU4F2 and AMID* before and after treatment with 5-Aza-2′-deoxycytidine in ALL cell lines. **B.** Pyrosequencing analysis of *-miR-34b/c, POU4F1, POU4F2 and AMID* before and after treatment with 5-Aza-2′-deoxycytidine in ALL cell lines. X axis shows the different loci analyzed. **C.** Q-RT-PCR analysis of *hsa-miR-34b/c, POU4F1, POU4F2 and AMID* before and after treatment with 5-Aza-2′-deoxycytidine in ALL cell lines. AZA: 5-Aza-2′-deoxycytidine. PB: Peripheral Blood sample.
(TIF)

Table S1 **Clinical characteristics and outcome of 200 ALL patients according to gene methylation status.**
(DOC)

Table S2 **Primers corresponding to methylated reactions of genes.**
(DOC)

Table S3 **Primers corresponding to unmethylated reactions of genes.**
(DOC)

Table S4 **Specific primers and probes corresponding to pyrosequencing analysis.**
(DOC)

Table S5 **Beta value and genes differentially methylated in more than 10% of ALL samples.** Different subtypes of leukemia or controls are color-coded. PB: Peripheral Blood mononuclear cells from healthy donors; CD3: CD3+ cells from PB and CD19: CD19+ cells from PB. PcG: targets of polycomb repressive complex 2 (PRC2). Light Red: genes hypermethylated in more than 10% of ALL samples. Light Green: genes hypomethylated in more than 10% of ALL samples.
(XLS)

Table S6 **Multivariate Cox Model for Disease Free Survival (DFS).**
(DOC)

Table S7 **Multivariate Cox Model for Overall Survival (OS).**
(DOC)

Table S8 **Multivariate Cox Model for Event-free survival (EFS).**
(DOC)

Table S9 **Specific primers and probes corresponding to Q-RT-PCR analysis.**
(DOC)
