# Supplementary figures and images for: Frequent and Simultaneous Epigenetic Inactivation of TP53 Pathway Genes in Acute Lymphoblastic Leukemia
Source: PLoS One. 2011 Feb 28;6(2):e17012. doi: 10.1371/journal.pone.0017012 (PMC3046174; doi:10.1371/journal.pone.0017012)

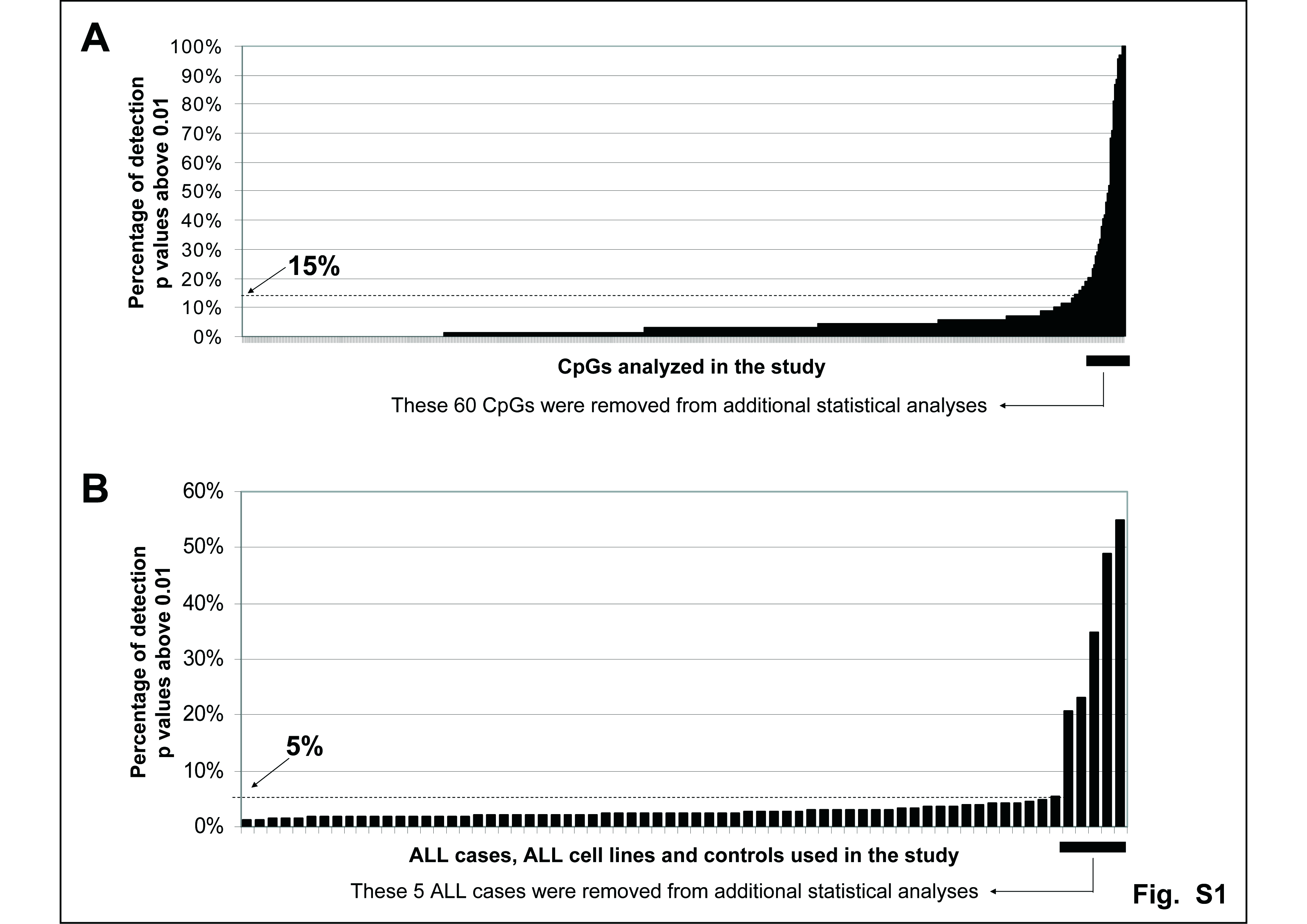

Supplement: Figure S1 — Definition of methylation analysis thresholds based on the distribution of bad detection p values (p>0.01). A) Threshold definition per CpG. A total of 1505 CpGs were studied with the bead array. The threshold of 15% was set based on visual inspection of this distribution (i.e. the estimated inflexion point). Based on this threshold, a total of 60 CpGs (the right peak of the plot) showed bad p values in 15% or more of the cases. B) Threshold definition per case. A total of 69 hybridizations were performed including ALLs and controls. As shown in the figure, low numbers of bad p values (ranging from 1 to 5%) are seen in the first 64 hybridizations. However, the 5 cases showed a clearly higher percentage of bad p values, Based on this distribution, we set a threshold for case selection of 5% (i.e. a case is selected for further statistical analyses if 95% or more CpGs show good p values). (TIF) [file pone.0017012.s001.tif]

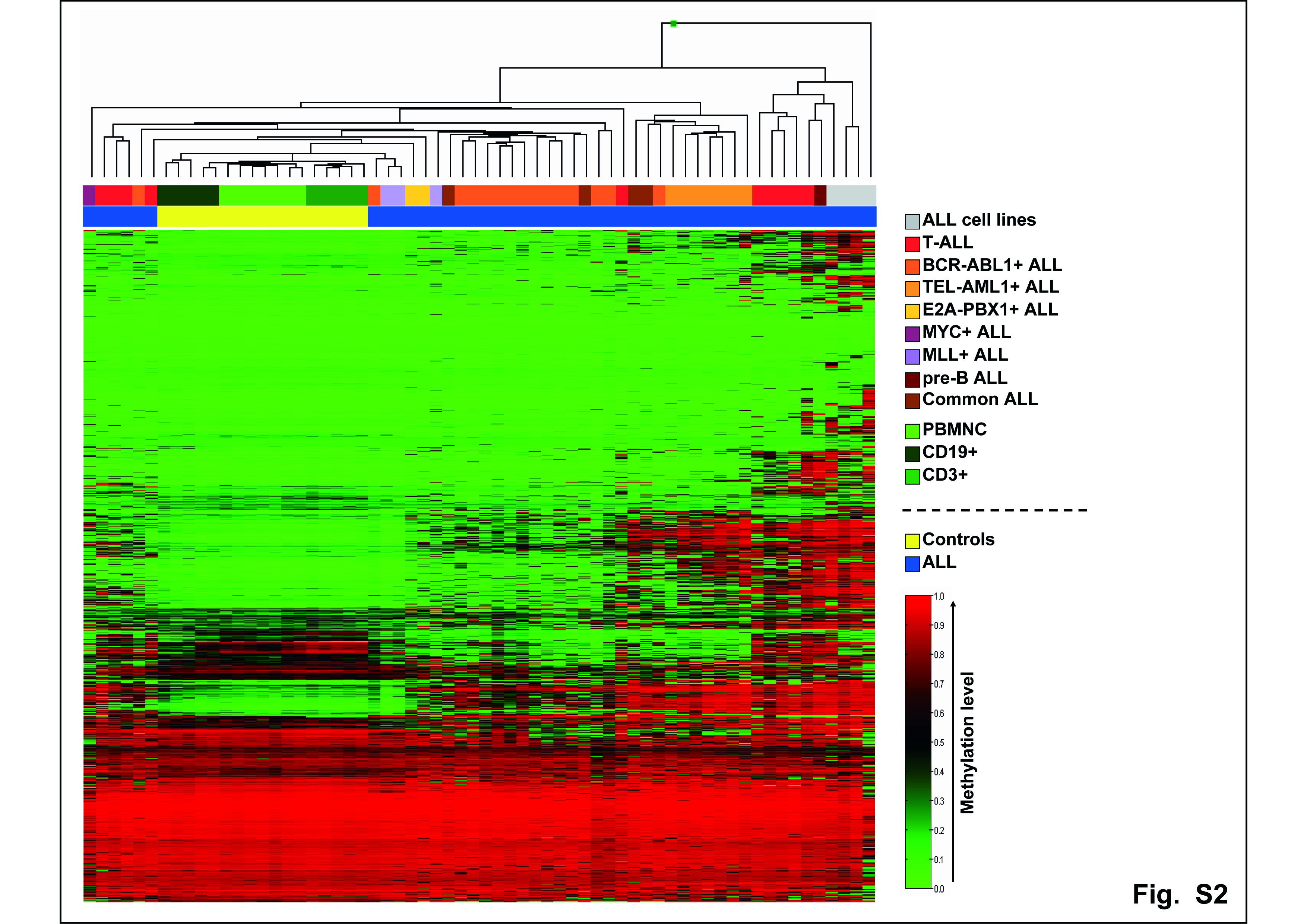

Supplement: Figure S2 — Hierarchical clustering analysis of DNA methylation data. Dendrogram of hierarchical cluster analysis based on the methylation status of CpG regions from ALL and control samples. The top bar beneath the dendrogram refers to the subtypes of ALL and specific controls from healthy donors, while the lower bar indicates ALL or control. Subtypes of leukemia or controls are color coded (ALL: Acute Lymphoblastic Leukemia; PBMNC: Peripheral Blood mononuclear cells from healthy donors, CD19+ cells from PB and CD3+ cells from PB). Red: methylated; Green: non-methylated. (TIF) [file pone.0017012.s002.tif]

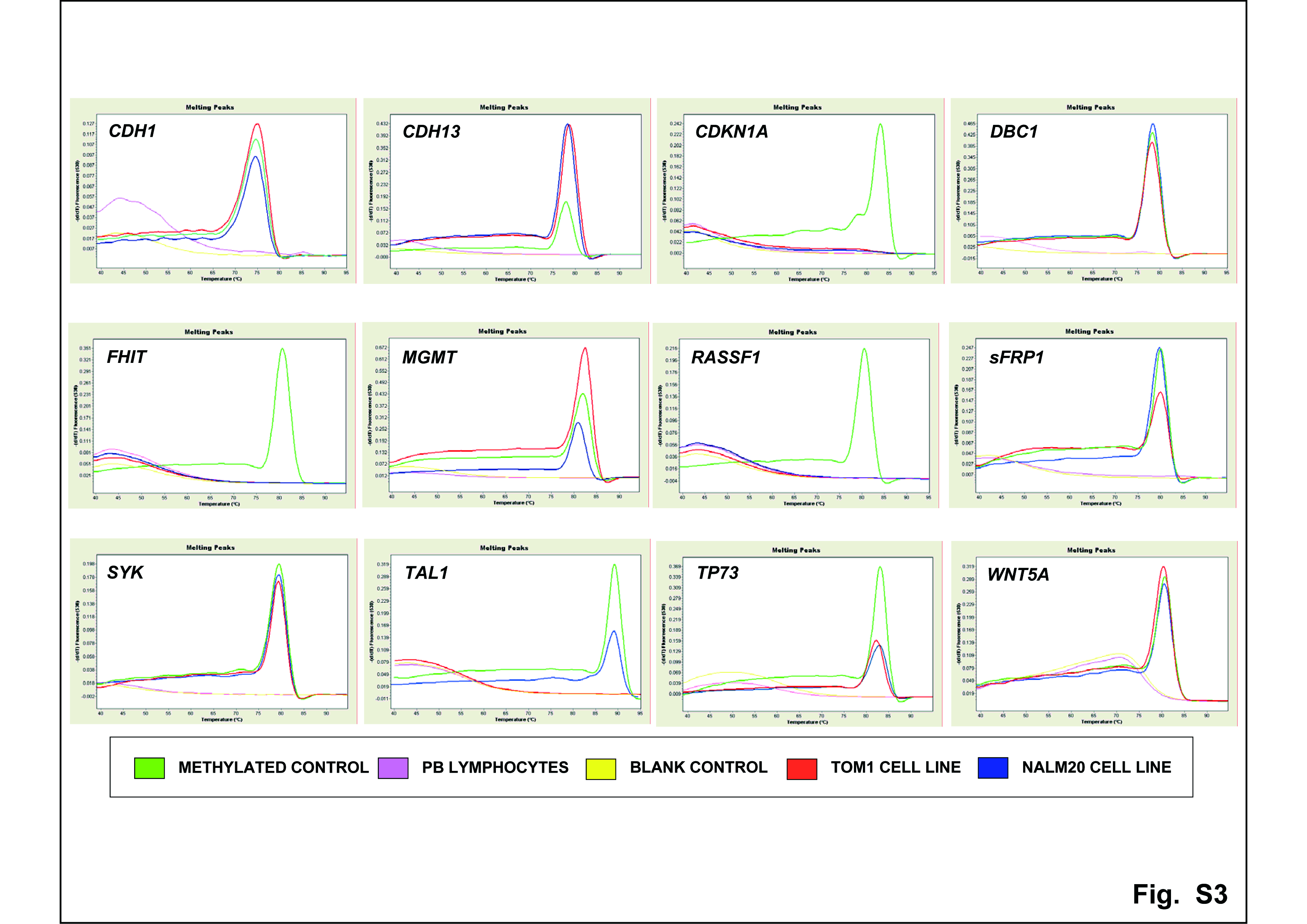

Supplement: Figure S3 — MSP melting curve analysis of the methylated sequences. The presence of specific methylated products is shown by the melting curves obtained by means of a LightCycler 2.0 PCR real time device. Green: positive methylated control; pink: peripheral blood lymphocytes from healthy donors; yellow: blank control (water); red: TOM1 cell line; blue: NALM-20 cell line. (TIF) [file pone.0017012.s003.tif]

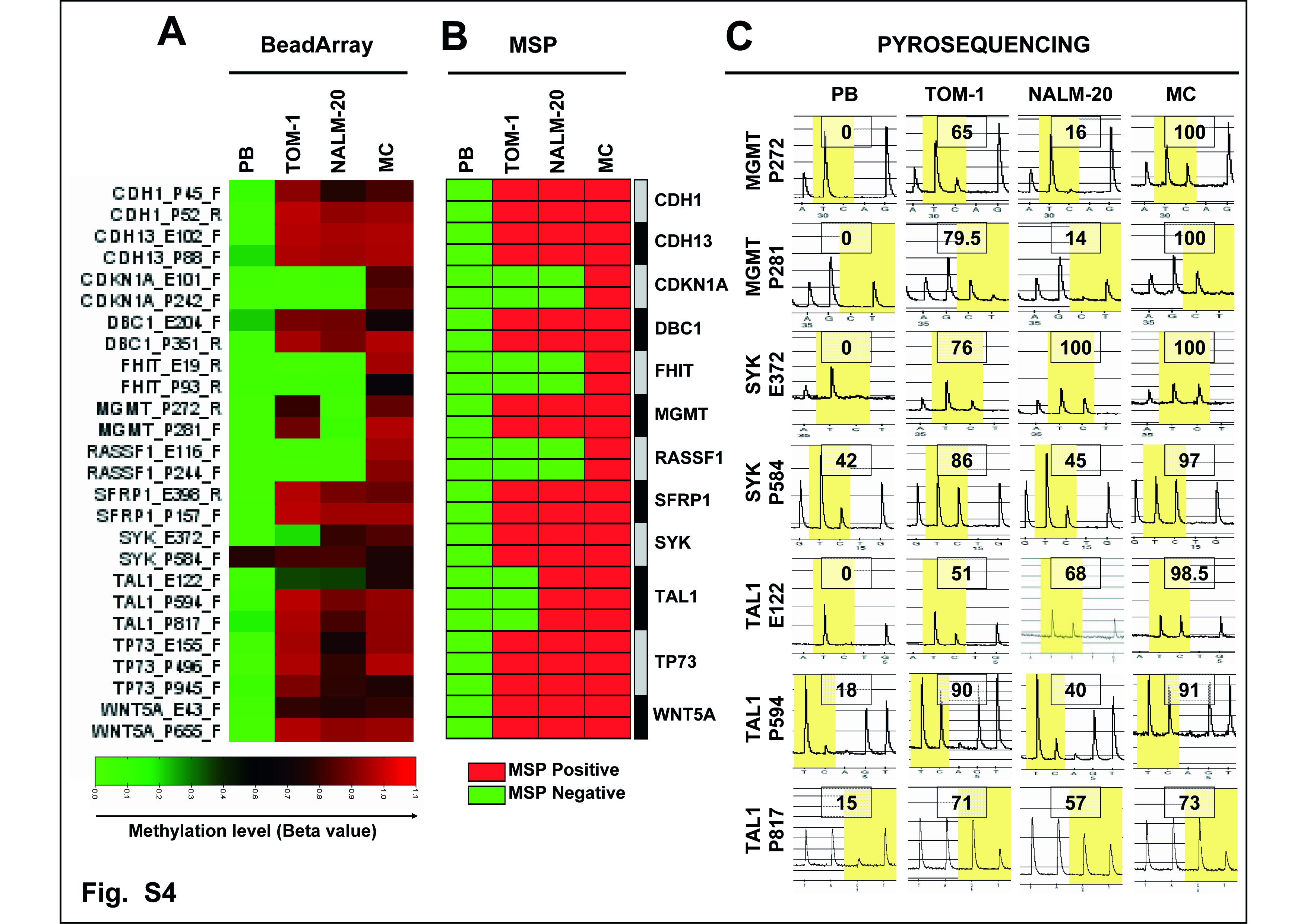

Supplement: Figure S4 — Comparison results obtained by Illumina Beadarray, Methylation Specific PCR (MSP) and pyrosequencing. A. Bead array methylation results. B. MSP results in same sample used in the bead array. Because several CpG for each gene on the array have been analyzed (A) and we have only one result of MSP (B), some of the MSP rows have been duplicated/triplicated to equal the number of CpGs. PB: Peripheral Blood of healthy donor, MC: human male genomic DNA universally methylated for all genes, Red: methylated, Green: non methylated. C. Pyrosequencing results of analyzed CpG loci on the array, corresponding to MGMT, SYK and TAL1 genes. The values are expressed as percentage of methylation. (TIF) [file pone.0017012.s004.tif]

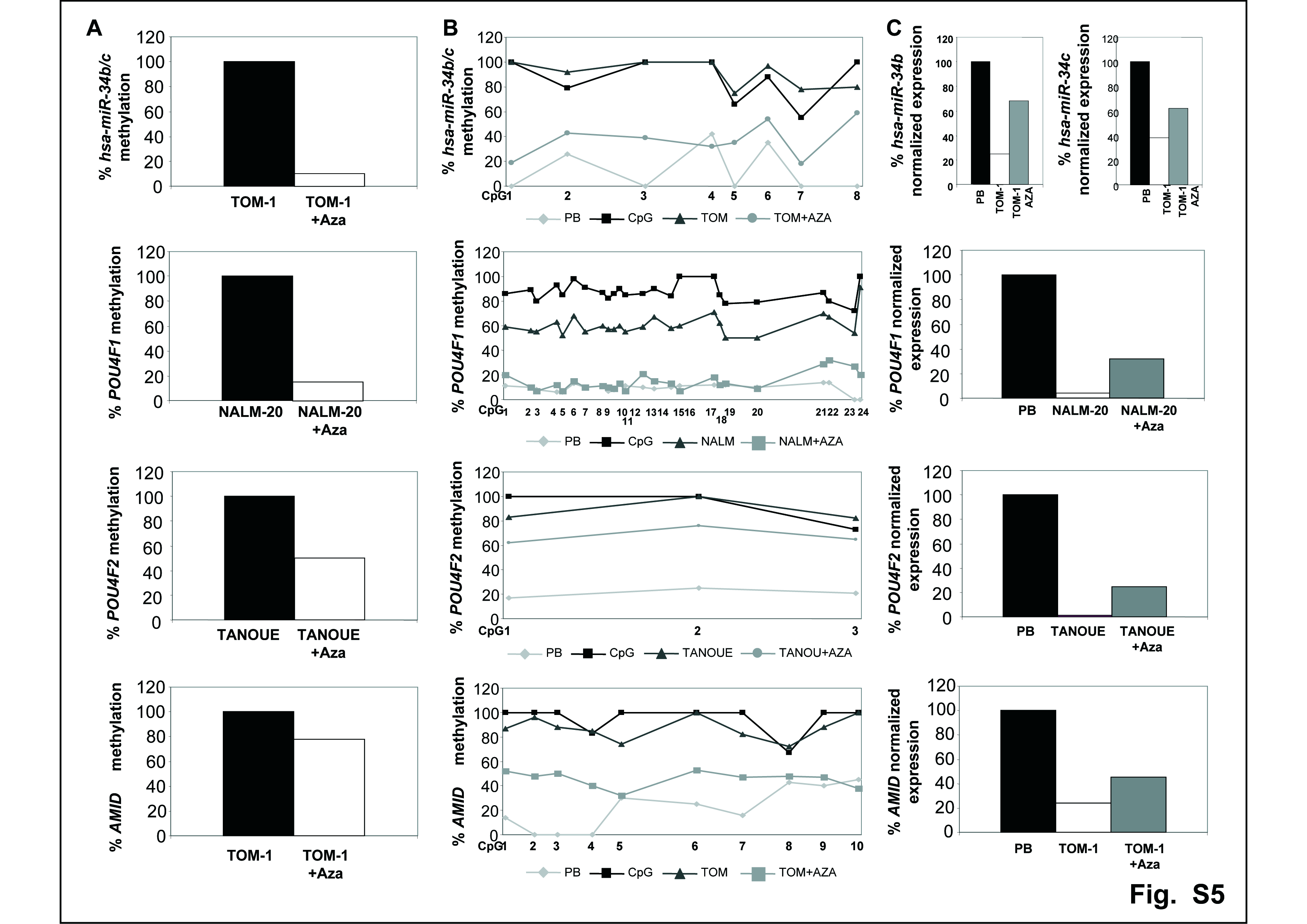

Supplement: Figure S5 — DNA methylation and expression analysis of hsa-miR-34b/c , POU4F1 , POU4F2 and AMID in ALL cell lines. A. Q-MSP analysis of hsa-miR-34b/c, POU4F1, POU4F2 and AMID before and after treatment with 5-Aza-2′-deoxycytidine in ALL cell lines. B. Pyrosequencing analysis of -miR-34b/c, POU4F1, POU4F2 and AMID before and after treatment with 5-Aza-2′-deoxycytidine in ALL cell lines. X axis shows the different loci analyzed. C. Q-RT-PCR analysis of hsa-miR-34b/c, POU4F1, POU4F2 and AMID before and after treatment with 5-Aza-2′-deoxycytidine in ALL cell lines. AZA: 5-Aza-2′-deoxycytidine. PB: Peripheral Blood sample. (TIF) [file pone.0017012.s005.tif]
