# Supplementary material for: Frequent and Simultaneous Epigenetic Inactivation of TP53 Pathway Genes in Acute Lymphoblastic Leukemia
Source: PLoS One. 2011 Feb 28;6(2):e17012. doi: 10.1371/journal.pone.0017012 (PMC3046174; doi:10.1371/journal.pone.0017012)
Supplement: Table S1 — Clinical characteristics and outcome of 200 ALL patients according to gene methylation status. (DOC) [file pone.0017012.s006.doc]

**SUPPLEMENTARY TABLE 1**

**Table S1:** Clinical characteristics and outcome of 200 ALL patients according to gene methylation status

| Feature | **Non-methylated (n=43)** | **Methylated**  **(n=157)** | ***P* value** |
| --- | --- | --- | --- |
| **Age** |  |  | **0.015** |
| < 15 years | 27 (63) | 64 (41) |  |
| > 15 years | 16 (37) | 93 (59) |  |
| **Sex (M/F)** | 23/20 (53/47) | 90/67 (57/43) | 0.604 |
| **WBC** |  |  | **0.05** |
| < 50 x 10^9^/L | 36 (84) | 103 (66) |  |
| > 50 x 10^9^/L | 7 (16) | 54 (34) |  |
| **FAB Classification** |  |  | **0.005** |
| L1 | 20 (47) | 37 (24) |  |
| L2 | 20 (47) | 114 (73) |  |
| L3 | 3 (6) | 6 (3) |  |
| **Blast Lineage** |  |  | **0.05** |
| B cell | 39 (91) | 119 (76) |  |
| T cell | 4 (9) | 38 (24) |  |
| **NCI Risk Groups** |  |  | 0.423 |
| Standard | 30 (70) | 100 (64) |  |
| High | 13 (30) | 57 (36) |  |
| **PETHEMA Risk Groups** |  |  | 0.558 |
| Standard | 28 (65) | 109 (69) |  |
| High | 15 (35) | 46 (31) |  |
| **Treatment** |  |  | 0.269 |
| PETHEMA 89 | 13 (30) | 35 (22) |  |
| PETHEMA 93 | 30 (70) | 122 (78) |  |
| BMT | 10 (23) | 32 (20) | 0.676 |
| **Best Response** |  |  |  |
| CR | 39 (91) | 133 (84) | 0.339 |
| Cytogenetic/Molecular Abnormalities |  |  |  |
| BCR-ABL1 | 11 (26) | 38 (24) | 0.843 |
| t(1;19) | 0 (0) | 2 (1) | 0.754 |
| 11q23 | 0 (0) | 2 (1) | 0.695 |
| c-myc | 0 (0) | 3 (2) | 0.487 |
| 7q35-14q11 | 0 (0) | 8 (5) | 0.236 |
| Hyperdiploidy | 3 (7) | 8 (5) | 0.589 |
| TEL-AML1 | 7 (16) | 24 (15) | 0.807 |
| Normal | 21 (49) | 69 (44) | 0.712 |
| Others | 1 (2) | 0 (0) | 0.898 |
| NT | 0 (0) | 3 (2) | 0758 |
| Relapse | 9 (21) | 73 (46) | **0.001** |
| BM relapse | 7 (16) | 62 (39) | **0.001** |
| CNS relapse | 2 (5) | 11 (7) | 0.357 |
| Death | 8 (19) | 88 (56) | **< 0.001** |
| Death in remission | 2 (4) | 5 (3) | 0.678 |

Data are expressed as counts and percentages (in parentheses). WBC indicates white blood count; FAB, French-American-British; NCI, National Cancer Institute; PETHEMA, *“Programa para el estudio y tratamiento de las hemopatias malignas”*; BMT, bone marrow transplantation; CR, complete remission. BM, bone marrow; CNS, central nervious system; NT, non-tested.
