# Supplementary material for: Frequent and Simultaneous Epigenetic Inactivation of TP53 Pathway Genes in Acute Lymphoblastic Leukemia
Source: PLoS One. 2011 Feb 28;6(2):e17012. doi: 10.1371/journal.pone.0017012 (PMC3046174; doi:10.1371/journal.pone.0017012)
Supplement: Table S2 — Primers corresponding to methylated reactions of genes. (DOC) [file pone.0017012.s007.doc]

**SUPPLEMENTARY TABLE 2**

**Table S2:** Primers corresponding to methylated reactions of genes

| **PRIMERS** | **SEQUENCE** | **AMPLIF.** | **Tª** |
| --- | --- | --- | --- |
| AMID-MF | TGTTTTAAAGTTTAAGGTCGTTCGT | 178 bp | 56ºC |
| AMID-MR | GAATTACTAACCCGAAACGCT |
| POU4F1-MF | GTTATTATAAGAGCGGTTTTTACGC | 125 bp | 58ºC |
| POU4F1-MR | GCTAACAAATATCAACTATCTCCGT |
| POU4F2-MF | GAGTCGTATGCGTATAGACGATTC | 176 bp | 58ºC |
| POU4F2-MR | AATACGAACGAAAATCGACGT |
| hsa-miR-34-MD | GTTCGTTGGTTTAGTTACGC | 179 bp | 55ºC |
| hsa-miR-34-MR | GACTACAACTCCCGAACGAT |
| TP73-MD | GCGTTCGGTTCGTAGGTT | 212 bp | 58ºC |
| TP73-MR | CTCAACTCCCAAAACCCAA |
| CDKN1C-MD | CGCGGTCGTTAATTAGTCGC | 263 bp | 54ºC |
| CDKN1C-MR | ACACAACGCACTTAACCTATAA |
| LATS2-MD | GTTTTAGATTCGAAAGGTCGTAGC | 191 bp | 57ºC |
| LATS2-MR | AAAACTAATTAACCCGTAAAACGAT |
| ASPP1-MD | AAGTAGGTGTTAGTTAAGGGCGTC | 205 bp | 55ºC |
| ASPP1-MR | ACCGACTATAAACCGAAAACGT |
| ASPP2-MD | ATTTTGTTTACGCGTTTAACGT | 164 bp | 56ºC |
| ASPP2-MR | GAAACAATTAAAAAACCGCG |
| CDKN1A-MD | GTGTTCGTTTTTGTAGTACGCG | 249 bp | 58ºC |
| CDKN1A-MR | GACTCCACAAAAAACTAACTTCGAC |
| P14-MD | GTCGAGTTCGGTTTTGGAGG | 164 bp | 59ºC |
| P14-MR | AAAACCACAACGACGAACG |
| CASP2-MD | AATTAATCGGATTTTTAGGTCGAAC | 157 bp | 58ºC |
| CASP2-MR | AACGCAAAACTCTAACGACGA |
| MDM2-MD | GTAACGGTTAAAGGAGTGTTATAGC | 148 bp | 55ºC |
| MDM2-MR | TCCGAAATAATAAAATAAAAAATATCG |
| RB1-MD | GTTTTAGTTTTTTATAGACGTC | 278 bp | 57ºC |
| RB1-MR | CGAAAAATTTTAAACGACATAA |
| RPS27L-MD | TTTAGAGTTAATCGAGAGCGG | 196 bp | 57ºC |
| RPS27L-MR | CCGTAAATCTAACGAACTTATTAACG |
| TIP60-MD | TTGATTTTCGGTAATATTTTTCGTC | 156 bp | 58ºC |
| TIP60-MR | ATAACGATCTACTAAATCCGTCACG |
| NOXA-MD | GTTTTTATTTTAGGACGCGTATTC | 232 bp | 56ºC |
| NOXA-MR | GAAAACTTTATACGCCCGAC |
| BBC3-MD | GTTTTTCGTTGTAGGGAAATTTTC | 276 bp | 57ºC |
| BBC3-MR | GTCGTAACCGCTACTAAAATCG |
| DBC1-MD | ATAGAGAGACGCGTAGATATAAACG | 269 bp | 57ºC |
| DBC1-MR | CCGAATAAACTAAAACTAAACCGTA |
| DAPK1-MD | GGATAGTCGGATCGAGTTAACGTC | 98 bp | 59ºC |
| DAPK1-MR | CCCTCCCAAACGCCG |
| BAX-MD | GATTAAATTTTTCGAGGGAGC | 117 bp | 57ºC |
| BAX-MR | GTAAAAACCCCGCTAAACGTA |
| CASP3-MD | GTAGTGTAGACGCGGTTTTTAGC | 164 bp | 59ºC |
| CASP3-MR | AAACCGAACAAATACCCGAA |
| APAF1-MD | TTTCGGGTAAAAGGGATAGAATTAGA | 140 bp | 56ºC |
| APAF1-MR | TATAACGCCCTTCCCCCGACGACG |
