# Supplementary material for: Frequent and Simultaneous Epigenetic Inactivation of TP53 Pathway Genes in Acute Lymphoblastic Leukemia
Source: PLoS One. 2011 Feb 28;6(2):e17012. doi: 10.1371/journal.pone.0017012 (PMC3046174; doi:10.1371/journal.pone.0017012)
Supplement: Table S3 — Primers corresponding to unmethylated reactions of genes. (DOC) [file pone.0017012.s008.doc]

**SUPPLEMENTARY TABLE 3**

**Table S3:** Primers corresponding to unmethylated reactions of genes

| **PRIMERS** | **SEQUENCE** | **AMPLIF.** | **Tª** |
| --- | --- | --- | --- |
| AMID-UF | TGTTTTAAAGTTTAAGGTTGTTTGT | 181 bp | 54ºC |
| AMID-UR | CCCAAATTACTAACCCAAAACACT |
| POU4F1-UD | GTTATTATAAGAGTGGTTTTTATGTGT | 125 bp | 53ºC |
| POU4F1-UR | ACTAACAAACATCAACTATCTCCATC |
| POU4F2-UD | AGTTGTATGTGTATAGATGATTTGA | 177 bp | 52ºC |
| POU4F2-UR | CCAATACAAACAAAAATCAACATT |
| hsa-miR-34-UD | GTTTGTTGGTTTAGTTATGTGT | 180 bp | 51ºC |
| hsa-miR-34-UR | CAACTACAACTCCCAAACAAT |
| TP73-UD | GTAGGTTTAGGGGTGTGTTTTGT | 166 bp | 51ºC |
| TP73-UR | CAAAAATATCAAAATCTACACAAT |
| CDKN1C-UD | TGTGGTTGTTAATTAGTTGT | 263 bp | 48ºC |
| CDKN1C-UR | ACACAACACACTTAACCTATAA |
| LATS2-UD | GGTGTTTTAGATTTGAAAGGTTGTAGT | 196 bp | 55ºC |
| LATS2-UR | AAAAAACTAATTAACCCATAAAACAAT |
| ASPP1-UD | AAGTAGGTGTTAGTTAAGGGTGTTG | 205 bp | 56ºC |
| ASPP1-UR | AAACCAACTATAAACCAAAAACATC |
| ASPP2-UD | ATTTTGTTTATGTGTTTAATGTTGG | 165 bp | 54ºC |
| ASPP2-UR | CAAAACAATTAAAAAACCACACC |
| CDKN1A-UD | TGTGTTTAGTGTATTAATGTAGGTGA | 295 bp | 54ºC |
| CDKN1A-UR | AACTCCACAAAAAACTAACTTCAAC |
| P14-UD | TTGGTGATTTTTTGGATTTGG | 170 bp | 55ºC |
| P14-UR | CTAAAAAACAACTACTACCCTAAACACT |
| CASP2-UD | TTAATTGGATTTTTAGGTTGAATGG | 157 bp | 55ºC |
| CASP2-UR | CAAACACAAAACTCTAACAACAAC |
| MDM2-UD | AATGGTTAAAGGAGTGTTATGTGT | 140 bp | 54ºC |
| MDM2-UR | ACCTCCAAAATAATAAAATAAAAAATATCA |
| RB1-UD | TTTAGTTTTTTATAGATGTTGGTGG | 275 bp | 54ºC |
| RB1-UR | AAAAAATTTTAAACAACATAACACC |
| RPS27L-UD | ATTTTTAGAGTTAATTGAGAGTGGTTG | 200 bp | 57ºC |
| RPS27L-UR | CCCATAAATCTAACAAACTTATTAACAAA |
| TIP60-UD | GATTTTTGGTAATATTTTTTGTTGT | 161 bp | 54ºC |
| TIP60-UR | AATAACAATCTACTAAATCCATCACATA |
| NOXA-UD | GGTTTTTATTTTAGGATGTGTATTTGG | 235 bp | 53ºC |
| NOXA-UR | CCAAAAACTTTATACACCCAAC |
| BBC3-UD | GTTTTTTGTTGTAGGGAAATTTTTG | 321 bp | 58ºC |
| BBC3-UR | TATAACTATAACTACTACTACTCCCCAAACCA |
| DBC1-UD | ATAGAGAGACGCGTAGATATAAACG | 269 bp | 56ºC |
| DBC1-UR | CCGAATAAACTAAAACTAAACCGTA |
| DAPK1-UD | GGAGGATAGTTGGATTGAGTTAATGTT | 106 bp | 60ºC |
| DAPK1-UR | CAAATCCCTCCCAAACACCAA |
| BAX-UD | GTAGTGTAGATGTGGTTTTTAGTGG | 166 bp | 56ºC |
| BAX-UR | CCAAACCAAACAAATACCCAA |
| CASP3-UD | ATTTAGATTTTAAATTTGGGGTGG | 277bp | 58ºC |
| CASP3-UR | CACCAAAAAATATACTCCAACACC |
| APAF1-UD | GATTTGAGGTGTTGTAGTGGTATTT | 100 bp | 56ºC |
| APAF-1 UR | AAAAAAAATCTTCCCAACCTATAACA |
