# Supplementary material for: Frequent and Simultaneous Epigenetic Inactivation of TP53 Pathway Genes in Acute Lymphoblastic Leukemia
Source: PLoS One. 2011 Feb 28;6(2):e17012. doi: 10.1371/journal.pone.0017012 (PMC3046174; doi:10.1371/journal.pone.0017012)
Supplement: Table S4 — Specific primers and probes corresponding to pyrosequencing analysis. (DOC) [file pone.0017012.s009.doc]

**SUPPLEMENTARY TABLE 4**

**Table S4:** Specific primers and probes corresponding to pyrosequencing analysis

| **PRIMERS** | **SEQUENCE** | **AMPLIF.** | **Tª** | **INTERMEDIATE PYROSEQUENCING PRIMERS** |
| --- | --- | --- | --- | --- |
| MGMT-D | TGGTAAATTAAGGTATAGAGTTTTAGG | 295 bp | 54ºC | GAAGTTGGGAAGG |
| MGMT-R | AAAACCTAAAAAAAACAAAAAAAC |
| SYK-D1 | AAAATAGGTATTGTTAATTAGTGTTATTAA | 205 bp | 53ºC | GGTTTAATTTATTTGGTTGTG |
| SYK-R1 | AAACTTTACTTCCTTCTTACCATAAC |
| SYK-D2 | TTATGTGAATAAAATATAGGTGGGTTT | 269 bp | 54ºC | GGAGAGAGGGGTTTAGGTTT |
| SYK-R2 | AAAAAAAATATTCTAACTCCAAAATC |
| TAL-D1 | GTATTGTATTGTAATTTAGGGAAGAATTAT | 243 bp | 53ºC | GTAAATAGAAGGAGGTTT |
| TAL-R1 | ACAACAATAAACAAAATAAAAAAAA |
| TAL-D2 | GGAGATTTGGTTGAGGAGGTAAT | 234 bp | 59ºC | ATTTTAGTTGGAGGGT |
| TAL-R2 | CCCCCAACCTACAAATATTTAAAA |
| TAL-D3 | TAGTTTGTTTTTTTTGGTTTTTTTG | 143 bp | 52ºC | GGAAGAGAAAATGGAGAGAG |
| TSL-R3 | TTTACCTTATAACCAAATCTCTATATCC |
| POU4F1-D | TGGATTTTTTGATTTTTTAGATTA | 306 bp | 55ºC | TTTGTTGGTGGTTATTATAA |
| POU4F1-R | AAATACACTCCTCTAACACCTAAACC |
| POU4F2-D | AGAGAAAAGGGGGGTAAGAGG | 363 bp | 59ºC | TGTTGTGGGAGGGGTGGGGA |
| POU4F2-R | ACTCAAATAACCCTAATCCCAAATT |
| AMID-D | GAGTATTTGTTTTAAAGTTTAAGGT | 476 bp | 51ºC | AGTGTTGAGTTA |
| AMID-R | ACCAAAAACACATCTCCCTATTAC |
| miR-34b/c-D | GTTTGGTATTTTTGGGGGTTAT | 312 bp | 56ºC | GTGAAATGGGGT |
| miR-34b/c-R | ACCACAATACAATCAACTAATA |
| DBC1-D | GAGAAATAAATAGTGTTAAA | 185 bp | 44ºC | GAGATAAAATTTATATT |
| DBC1-R | AAATCCTAATACCCTTAAA |
