# Supplementary material for: Frequent and Simultaneous Epigenetic Inactivation of TP53 Pathway Genes in Acute Lymphoblastic Leukemia
Source: PLoS One. 2011 Feb 28;6(2):e17012. doi: 10.1371/journal.pone.0017012 (PMC3046174; doi:10.1371/journal.pone.0017012)
Supplement: Table S6 — Multivariate Cox Model for Disease Free Survival (DFS). (DOC) [file pone.0017012.s011.doc]

**SUPPLEMENTARY TABLE 6**

**Table S6: Multivariate Cox Model for Disease Free Survival (DFS).**

| **Feature** | **Univariate Analysis** | | **Multivariate Analysis** | |
| --- | --- | --- | --- | --- |
|  | **P** | **Hazard ratio (95% CI)** | **P** | **Hazard ratio (95% CI)** |
| **Global Series (n = 200)** |  |  |  |  |
| Methylation profile | 0.008 | 2.613 (1.286-5.309) | 0.006 | 2.699 (1.332-5.472) |
| WBC count | 0.048 | 1.733 (1.044-2.878) | 0.152 | 1.363 (0.807-2.303) |
| BCR-ABL1 | < 0.001 | 2.973 (1.756-5.033) | < 0.001 | 3.034 (1.843-4.993) |
| Immunophenotype | 0.100 | 2.051 (0.795-5.294) | 0.849 | 1.030 (0.552-1.924) |
| Age | 0.001 | 2.805 (1.532-5.124) | 0.001 | 2.978 (1.801-5.012) |
| PETHEMA risk groups | 0.050 | 1.589 (0.973-2.596) | 0.425 | 1.321 (0.781-2.234) |
|  |  |  |  |  |
| Chilhood ALL (n= 91) |  |  |  |  |
| Methylation profile | 0.010 | 2.395 (1.160-4.947) | 0.050 | 2.492 (1.235-4.732) |
| NCI risk groups | 0.080 | 1.670 (0.767-3.639) | 0.796 | 1.202 (0.612-3.365) |
| Immunophenotype | 0.080 | 1.865 (0.915-3.799) | 0.292 | 1.543 (0.871-3.233) |
| WBC count | 0.100 | 1.729 (0.867-3.449) | 0.845 | 1.150 (0.734-3.512) |
|  |  |  |  |  |
| **Adult ALL (n= 109)** |  |  |  |  |
| Methylation profile | 0.019 | 2.756 (0.966-7.868) | 0.050 | 2.977 (1.803-5.015) |
| WBC count | 0.041 | 1.802 (0.932-3.484) | 0.284 | 1.583 (0.970-2.587) |
| BCR-ABL1 | < 0.001 | 3.910 (2.040-7.494) | < 0.001 | 4.439 (2.463-7.998) |

Multivariate Cox regression modelling was performed for DFS using a forward-selection stepwise modeling process (with a forward selection method with entry probability of *P* = .01. using Wald CIs and with stepwise removal of nonsignificant factors); the difference in the log likelihood (−2×log likelihood) was used. Factors were entered as categorical values. The following variables were considered in the model: age (≤ 15 vs. > 15 years), methylation profile (negative vs. positive), WBC count (≤ 50x10^9^/l vs. > 50x10^9^/l), BCR-ABL (negative vs positive), cell immunophenotype (B vs. T) and PETHEMA risk groups (high vs. others). For children we also included TEL-AML1 (positive vs. negative) and NCI risk groups (high vs. others). Each variable listed was adjusted for all of the others.
