# Supplementary material for: Frequent and Simultaneous Epigenetic Inactivation of TP53 Pathway Genes in Acute Lymphoblastic Leukemia
Source: PLoS One. 2011 Feb 28;6(2):e17012. doi: 10.1371/journal.pone.0017012 (PMC3046174; doi:10.1371/journal.pone.0017012)
Supplement: Table S7 — Multivariate Cox Model for Overall Survival (OS). (DOC) [file pone.0017012.s012.doc]

**SUPPLEMENTARY TABLE 7**

**Table S7: Multivariate Cox Model for Overall Survival (OS).**

| **Feature** | **Univariate Analysis** | | **Multivariate Analysis** | |
| --- | --- | --- | --- | --- |
|  | **P** | **Hazard ratio (95% CI)** | **P** | **Hazard ratio (95% CI)** |
| **Global Series (n = 200)** |  |  |  |  |
| Methylation profile | < 0.001 | 3.120 (1.426-6.827) | 0.005 | 3.089 (1.411-6.760) |
| WBC count | 0.005 | 1.725 (0.986-2.243) | 0.570 | 0.844 (0.471-1.511) |
| BCR-ABL1 | < 0.001 | 2.861 (1.763-4.642) | < 0.001 | 3.087 (1.963-4.845) |
| Immunophenotype | 0.1000 | 1.444 (0.930-2.243) | 0.475 | 0.878 (0.546-1.746) |
| Age | < 0.001 | 3.190 (1.356-5.321) | < 0.001 | 3.054 (1.412-5.212) |
| PETHEMA risk groups | 0.08 | 1.744 (0.725-4.196) | 0.102 | 1.426 (0.899-4.261) |
|  |  |  |  |  |
| Chilhood ALL (n = 91) |  |  |  |  |
| Methylation profile | 0.026 | 7.040 (0.898-55.190) | 0.050 | 7.122 (0.940-53.934) |
| NCI risk groups | 0.100 | 2.932 (0.812-14.211) | 0.426 | 2.159 (0.901-13.898) |
| Immunophenotype | 0.100 | 3.261 (0.725-14.662) | 0.398 | 2.168 (0.731-14.569) |
| WBC count | 0.100 | 3.737 (0.992-14.075) | 0.879 | 1.915 (0.900-14.258) |
|  |  |  |  |  |
| **Adult ALL (n = 109)** |  |  |  |  |
| Methylation profile | 0.019 | 2.188 (0.932-5.139) | 0.050 | 2.632 (1.135-6.105) |
| WBC count | 0.011 | 1.538 (0.939-2.520) | 0.056 | 1845 (1.136-2.995) |
| BCR-ABL1 | < 0.001 | 3.012 (1.792-5.061) | < 0.001 | 3.899 (2.391-6.356) |

Multivariate Cox regression modeling was performed for OS using a forward-selection stepwise process (with a forward selection method with entry probability of *P* = .01. using Wald CIs and with stepwise removal of non-significant factors); the difference in the log likelihood (−2×log likelihood) was used. Factors were entered as categorical values. The following variables were considered in the model: age (≤ 15 vs. > 15 years), methylation profile (negative vs. positive), WBC count (≤ 50x10^9^/l vs. > 50x10^9^/l), BCR-ABL (negative vs. positive), cell immunophenotype (B vs. T) and PETHEMA risk groups (high vs. others). For children we also included TEL-AML1 (positive vs. negative) and NCI risk groups (high vs. others). Each variable listed was adjusted for all of the others.
