# Supplementary material for: Frequent and Simultaneous Epigenetic Inactivation of TP53 Pathway Genes in Acute Lymphoblastic Leukemia
Source: PLoS One. 2011 Feb 28;6(2):e17012. doi: 10.1371/journal.pone.0017012 (PMC3046174; doi:10.1371/journal.pone.0017012)
Supplement: Table S8 — Multivariate Cox Model for Event-free survival (EFS). (DOC) [file pone.0017012.s013.doc]

**SUPPLEMENTARY TABLE 8**

**Table S8: Multivariate Cox Model for Event-free survival (EFS).**

| **Feature** | **Univariate Analysis** | | **Multivariate Analysis** | |
| --- | --- | --- | --- | --- |
|  | **P** | **Hazard ratio (95% CI)** | **P** | **Hazard ratio (95% CI)** |
| **Global Series (n = 200)** |  |  |  |  |
| Methylation profile | 0.008 | 2.284 (1.238-4.213) | 0.010 | 2.225 (1.207-4.101) |
| WBC count | 0.046 | 1.535 (1.007-2.340) | 0.040 | 1.548 (1.020-2.349) |
| BCR-ABL1 | < 0.001 | 2.469 (1.578-3.862) | < 0.001 | 2.582 (1.677-3.984) |
| Age | < 0.001 | 3.152 (1.341-5.182) | < 0.001 | 3.002 (1.408-5.196) |
|  |  |  |  |  |
| Chilhood ALL (n = 91) |  |  |  |  |
| Methylation profile | 0.094 | 2.305 (0.866-6.132) | 0.050 | 2.603 (0.999-6.783) |
|  |  |  |  |  |
| **Adult ALL (n = 109)** |  |  |  |  |
| Methylation profile | 0.100 | 1.885 (0.856-4.154) | 0.122 | 1.795 (0.872-4.032) |
| WBC count | 0.056 | 1.610 (0.987-2.625) | 0.062 | 1.711 (0.993-2.795) |
| BCR-ABL1 | < 0.001 | 2.964 (1.809-4.857) | < 0.001 | 3.445 (2.134-5.591) |

Multivariate Cox regression modeling was done for EFS using a forward-selection stepwise process (with a forward selection method with entry probability of *P* = .01. using Wald CIs and with stepwise removal of non-significant factors); the difference in the log likelihood (−2×log likelihood) was used. Factors were entered as categorical values. The following variables were considered in the model: age (≤ 15 vs. > 15 years), methylation profile (negative vs. positive), WBC count (≤ 50x10^9^/l vs. > 50x10^9^/l), BCR-ABL (negative vs. positive), cell immunophenotype (B vs. T) and PETHEMA risk groups (high vs. others). For children we also included TEL-AML1 (positive vs. negative) and NCI risk groups (high vs. others). Each variable listed was adjusted for all of the others.
