# Supplementary material for: Frequent and Simultaneous Epigenetic Inactivation of TP53 Pathway Genes in Acute Lymphoblastic Leukemia
Source: PLoS One. 2011 Feb 28;6(2):e17012. doi: 10.1371/journal.pone.0017012 (PMC3046174; doi:10.1371/journal.pone.0017012)
Supplement: Table S9 — Specific primers and probes corresponding to Q-RT-PCR analysis. (DOC) [file pone.0017012.s014.doc]

**SUPPLEMENTARY TABLE 9**

**Table S9:** Specific primers and probes corresponding to Q-RT-PCR analysis

| **PRIMERS** | **SEQUENCE** |
| --- | --- |
| AMID-F | GAGTGGAGATGGCAGCAGAGA |
| AMID-R | GTCAGCCAGGGCCACTTG |
| AMID-S | FAM-TAAAACAGAATATCCTGAGAAAGAGGTCACTC-TAMRA |
| POU4F1-F | CACTTTCCCGCGGACTTTC |
| POU4F1-R | GTCCAGGCTGGCGAAGAG |
| POUAF1-S | FAM-GAGTGTTTGTGGATATACATGCCAAGC-TAMRA |
| POU4F2-F | GCTATGCGGAGAGCCTGTCT |
| POU4F2-R | TGGGCACCGATGAAGAAGAG |
| POU4F2-S | FAM-GAATATATTCGGCGGGCTGGATGAGAGTC-TAMRA |
